# Towards the prediction of drug solubility in binary solvent mixtures at various temperatures using machine learning

**DOI:** 10.1186/s13321-024-00911-3

**Published:** 2024-10-28

**Authors:** Zeqing Bao, Gary Tom, Austin Cheng, Jeffrey Watchorn, Alán Aspuru-Guzik, Christine Allen

**Affiliations:** 1 Leslie Dan Faculty of Pharmacy, University of Toronto, Toronto, ON M5S 3M2 Canada; 2 Department of Chemistry, University of Toronto, Toronto, ON M5S 3H6 Canada; 3 Department of Computer Science, University of Toronto, Toronto, ON M5S 2E4 Canada; 4 Vector Institute for Artificial Intelligence, Toronto, ON M5S 1M1 Canada; 5 Acceleration Consortium, Toronto, ON M5S 3H6 Canada; 6 Lebovic Fellow, Canadian Institute for Advanced Research (CIFAR), Toronto, ON M5S 1M1 Canada; 7 Department of Chemical Engineering and Applied Chemistry, University of Toronto, Toronto, ON M5S 3E5 Canada; 8 Department of Materials Science and Engineering, University of Toronto, Toronto, ON M5S 3E4 Canada; 9 CIFAR Artificial Intelligence Research Chair, Vector Institute, Toronto, ON M5S 1M1 Canada

## Abstract

**Abstract:**

Drug solubility is an important parameter in the drug development process, yet it is often tedious and challenging to measure, especially for expensive drugs or those available in small quantities. To alleviate these challenges, machine learning (ML) has been applied to predict drug solubility as an alternative approach. However, the majority of existing ML research has focused on the predictions of aqueous solubility and/or solubility at specific temperatures, which restricts the model applicability in pharmaceutical development. To bridge this gap, we compiled a dataset of 27,000 solubility datapoints, including solubility of small molecules measured in a range of binary solvent mixtures under various temperatures. Next, a panel of ML models were trained on this dataset with their hyperparameters tuned using Bayesian optimization. The resulting top-performing models, both gradient boosted decision trees (light gradient boosting machine and extreme gradient boosting), achieved mean absolute errors (MAE) of 0.33 for LogS (S in g/100 g) on the holdout set. These models were further validated through a prospective study, wherein the solubility of four drug molecules were predicted by the models and then validated with in-house solubility experiments. This prospective study demonstrated that the models accurately predicted the solubility of solutes in specific binary solvent mixtures under different temperatures, especially for drugs whose features closely align within the solutes in the dataset (MAE < 0.5 for LogS). To support future research and facilitate advancements in the field, we have made the dataset and code openly available.

**Scientific contribution**

Our research advances the state-of-the-art in predicting solubility for small molecules by leveraging ML and a uniquely comprehensive dataset. Unlike existing ML studies that predominantly focus on solubility in aqueous solvents at fixed temperatures, our work enables prediction of drug solubility in a variety of binary solvent mixtures over a broad temperature range, providing practical insights on the modeling of solubility for realistic pharmaceutical applications. These advancements along with the open access dataset and code support significant steps in the drug development process including new molecule discovery, drug analysis and formulation.

**Graphical Abstract:**

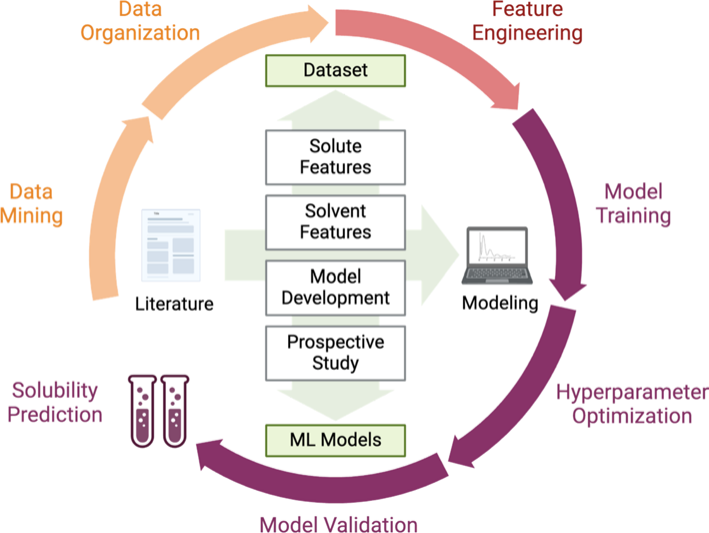

**Supplementary Information:**

The online version contains supplementary material available at 10.1186/s13321-024-00911-3.

## Introduction

In pharmaceutical development, the solubility of drugs is a critical factor that influences various stages, including drug discovery [1–3], drug analysis [2, 4, 5], and formulation design [6–8]. Drug solubility measurements primarily include thermodynamic and kinetic methods [9, 10]. Thermodynamic methods assess the solute concentration in a solution at equilibrium between dissolved and undissolved compounds, while kinetic methods identify the solute concentration at which precipitation first occurs [11]. Although both methods are effective and efforts have been made to enhance their throughput [1, 4, 12], they remain tedious and can pose challenges for costly drugs or when the available quantity of drug is limited. To address these challenges, researchers have begun to explore machine learning (ML) as an alternative method [13, 14]. As a data-driven approach, ML models can be trained on available solubility datasets to learn solute–solvent interactions for prediction of unmeasured solubility. To date, most of these ML models were developed to model the solute solubility in a specific solvent [15–25], with a primary focus on aqueous solubility due to availability of data for model development. Compared to models designed for predicting solubility across multiple solvents, these solvent-specific models generally deliver improved accuracy because their design does not require them to account for variations between different solvents. However, for the same reason, the applications of these solvent-specific models are limited to the specific solvents for which they were developed. To extend the applicability of these models, researchers have started to include a range of solvents into their datasets, allowing ML models to learn and adapt to different solvents [26–28]. A notable example is the work of Vassileiou et al., who compiled a dataset primarily from the literature, supplemented with in-house and industrial partner data [26]. This dataset includes 714 solubility data points measured at room temperature, covering 75 solutes and 49 solvents [26]. Using this dataset, ML models were trained to predict solubility across solvents, with the resulting model mean absolute error (MAE) ranging from 0.39 to 0.58 (LogS, S in g/100 g) [26]. Another significant contribution is from Ye et al., who collected a larger dataset of 5081 data points from the literature [27]. This dataset includes the solubility of 266 compounds in 123 solvents measured at various temperatures, and the optimal model resulted in predictions for solubility with an MAE of 0.47 (LogS, S in mol/L) [27].

Despite these significant advancements in predicting solubility within single solvents, the complicated nature of pharmaceutical research often necessitates an understanding of drug solubility in solvent mixtures [29–32]. These mixtures allow greater flexibility through adjusting solvent combinations and ratios, enabling solubility to be tailored to meet specific needs and to co-dissolve other necessary materials (e.g., excipients [33, 34]). However, the wide variety of possible solvent combinations/ratios and the influence of temperature significantly complicate the experimental measurements. This complexity further extends beyond experimental methods to ML approaches, which require extensive datasets covering a broad spectrum of these conditions for effective model training. The need for such an extensive dataset potentially contributes to the scarcity of research on modeling solubility in solvent mixtures with ML approaches. One of the very few examples in this field is work by Chinta et al. [35], who trained ML models to predict solubility in binary solvent mixtures. In that study, models were trained and evaluated using a relatively small dataset, containing around 600 solubility results for 27 solutes predominantly measured at a single temperature [35]. While that work makes for important progress, the limited size and scope of the dataset restrict the broader application of these models.

**Fig. 1:**
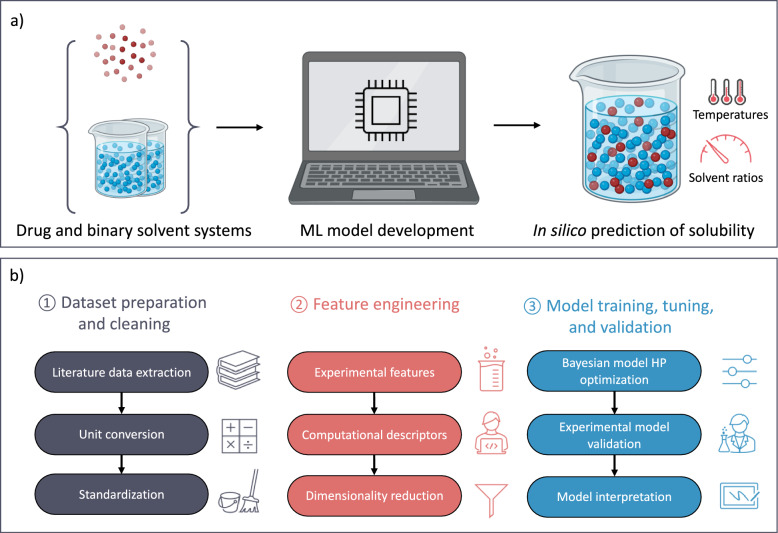
**a** Schematic outlining the study’s objective of utilizing ML approaches for predicting drug solubility in binary solvent mixtures. **b** Outline of the methodology deployed in this study. First, a comprehensive dataset was compiled through a literature review, followed by cleaning and standardization of the data. Next, both experimental and computational descriptors were incorporated into the dataset as input features, which were then refined to mitigate the risks associated with high-dimensional datasets. Lastly, this refined dataset was used to optimize the models’ hyperparameters (HPs), leading to identification of the top-performing models. Following its identification, these models were experimentally validated and then interpreted

To bridge the existing knowledge gap, we compiled an extensive dataset from the literature, consisting of 27,000 solubility data points measured in binary solvent mixtures at various temperatures (Fig. 1). The collected solubility studies were characterized by the conditions under which the measurements were made, as well as the experimental and computational features of the solutes and solvents. These features were then refined, with the goal of reducing the dimensionality of the dataset in terms of feature variance and correlation. Next, Bayesian hyperparameter optimization was applied to a selection of ML models to identify the most effective models and their optimal hyperparameter configurations. The identified models were further validated through a prospective study, demonstrating their potential for predicting drug solubility in specific binary solvent mixtures and at various temperatures. These models were particularly effective for drugs with properties well-represented within the dataset’s scope. The collected dataset and Python code are published for open access to facilitate future research in solubility modeling.

## Results

### Dataset overview

**Fig. 2:**
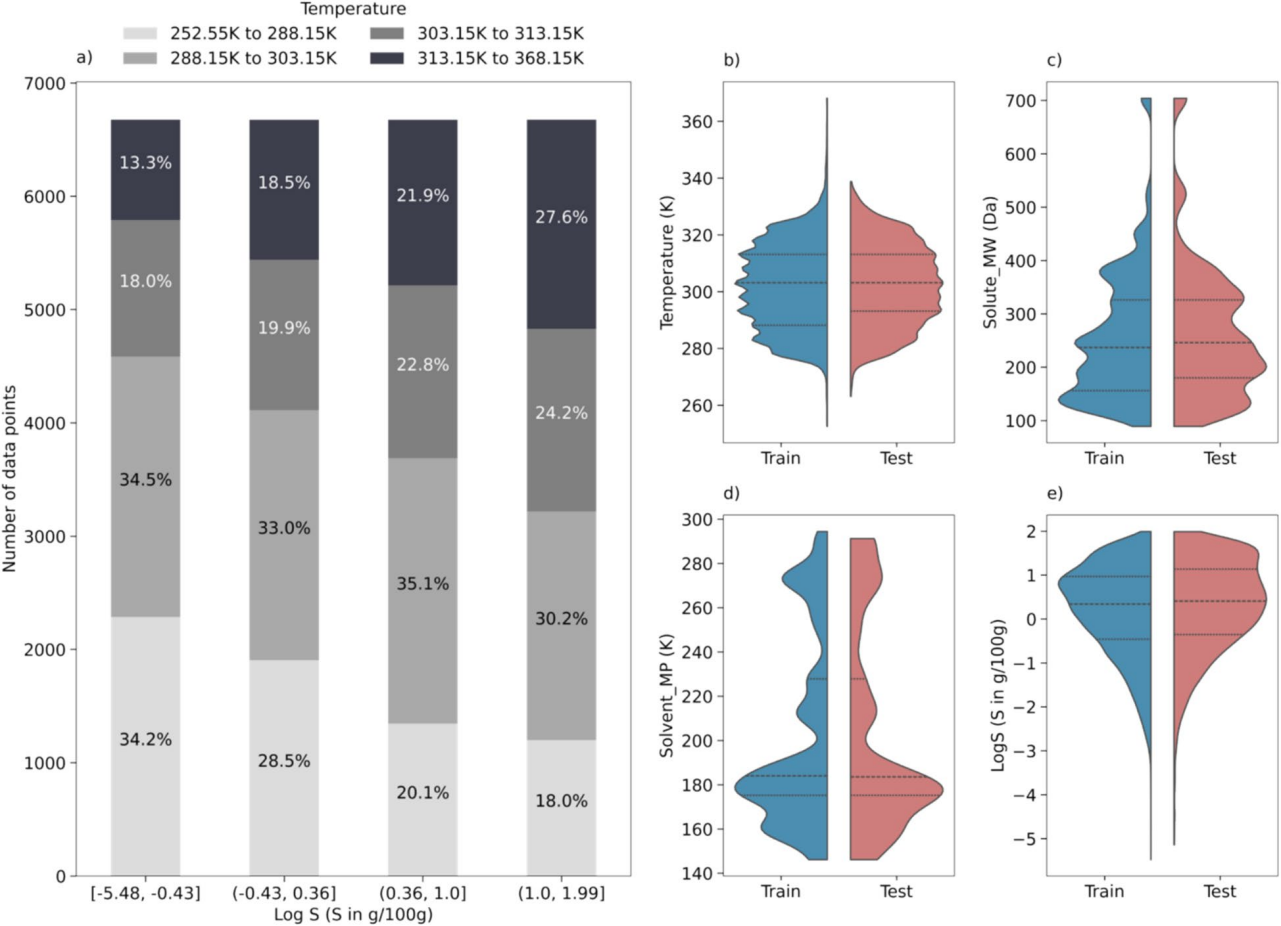
**a** Shows an overview of the dataset, illustrating the distribution of solubility values across the whole dataset. The bars are color-coded with a gradient to signify the range of investigation temperatures: lighter shades represent lower temperatures, while darker shades indicate higher temperatures. This dataset is divided into training and test subsets, with their investigation temperature, solute molecular weight (MW), solvent melting temperature (MP), and solubility distributions shown in violin plots (**b**–**e**). The dashed lines in the violin plots indicate the first, second, and third quartiles of the feature distribution

The dataset for this study was compiled through a literature review using the Web of Science database, with search keywords “solubility” and “binary system”. This literature search resulted in a dataset of 27,000 solubility data points, including 123 small-molecule solutes, 44 solvents, 110 binary solvent mixtures, and 373 unique solute-binary solvent systems. About 30% of the solutes are FDA-approved drugs [36]. The remaining compounds are mainly pharmaceutical intermediates, drug metabolites, and compounds with therapeutic potential. To the best of the authors’ knowledge, this is the most extensive open-access dataset on solubility in binary solvent systems available to date. Figure 2 provides an overview of the dataset. In Fig. 2a, the data points are categorized into quantiles based on their solubilities, which are reported in LogS (S in g/100 g) in this study. The color gradient of the bars represents specific temperature ranges, illustrating the effects of temperature on the solubility. As expected, higher temperatures are usually associated with increased solubility [37–39]. For example, in the lowest solubility quantile ([− 5.48, − 0.43]), only 13.3% of samples were recorded at higher temperatures (above 313.15 K). This proportion nearly doubles to 27.6% in the fourth quantile, which corresponds to higher solubility levels ([1.00, 1.99]).

The dataset was then divided into two distinct parts: 75% for the training set and 25% for the test set as described in the method section. The training set was used for feature engineering and model hyperparameter optimization, while the test set for model evaluation to identify the optimal models. Figure 2b to e show the distributions of four key attributes of solute–solvent systems including investigation temperature, solute molecular weight, solvent melting temperature, and reported solubility, across both subsets. These plots demonstrate that the test set generally aligns with the training set, evidenced by their comparable quantile values, ensuring that the model evaluation is representative and thorough.

### Feature engineering

Feature engineering is a critical step in the development of ML models, as it involves identifying the most informative features that can greatly improve accuracy by making it easier for models to learn the relationship between the inputs and the target [40–43]. For this study, the feature set initially collected from the literature was limited to solubility measurement parameters only, including solvent ratios and temperature of investigation. However, to predict the solubility in solvent mixtures, it was necessary to also include descriptors of the solute and the two solvents.

The selection of compound melting temperatures as a crucial experimental attribute was informed by the well-established effect of melting temperature on solubility, as noted in the literature [44–46], and its widespread availability from open access sources such as the Chemical Abstracts Service (CAS) database [47] and supplier websites. To ensure accuracy, each melting temperature was obtained from three different sources, and the average was used for modeling in this work. The melting temperatures from these various sources demonstrated high consistency, with a mean coefficient of variation (CV) of 1% and a maximum CV of 5%. In addition to collecting solute melting temperatures from public sources, separately we generated predicted solute melting temperatures using an open-access model [48] and included in the dataset. Our comparison of model performance, as detailed in Sect. "The effects of melting temperature on model performance”, when trained on published versus predicted solute melting temperatures aims to expand utility of the model to applications wherein molecules are not readily available for experimental determinations. Beyond melting temperatures, the dataset was enriched with computational descriptors for both solutes and solvents, incorporating MACCS molecular fingerprints and RDKit features to provide a comprehensive characterization. These features were generated by the RDKit package [49], a toolset for cheminformatics that processes molecular structures encoded in SMILES notation. A full list of these computational descriptors is included in Table S1.

However, the inclusion of the RDkit descriptors and MACCS fingerprints led to a substantial increase in dataset dimensionality, introducing over 1000 features for each solute-binary solvent system. High-dimensional data can pose challenges, such as overfitting, increased computational demands for model development, and difficulties in model interpretability [50–52]. To mitigate these challenges while maintaining dataset information, a standard feature refinement process based on feature variance and correlation was implemented. Initially, features demonstrating zero variance were removed, as their constant values across all observations provide no discriminative capability, thereby not enhancing the models’ predictive performance. Next, features exhibiting high correlation (Pearson correlation coefficient > 0.8 [53–55]) with others in the dataset were also removed to reduce redundancy, which can burden the model and diminish its ability to generalize. Through these steps, the dataset was condensed to 362 features, effectively reducing it to approximately one-third of its original volume. This refined dataset was utilized in subsequent studies.

Beyond these measures, principal component analysis (PCA) was also employed as an additional step for further feature reduction. PCA is another widely employed technique to manage high-dimensional data by transforming a large set of variables into a smaller number of principal components [56–59]. For instance, as illustrated in Figure S1, PCA was performed on the refined dataset (containing 362 features) and 85 principal component features were identified as capable of explaining the majority of the dataset’s variance (> 95%). However, employing PCA for dimensionality reduction comes with certain limitations, including challenges in model interpretability [60, 61] and the potential for dataset information loss during the transformation process [62, 63]. As shown in Figure S2, applying PCA for further feature reduction resulted in decreased model performance. Therefore, the dataset processed by PCA was not utilized further in this study.

### ML model development and evaluation

Using the refined dataset, a selection of ML models was trained and finetuned on the training set before their performance was assessed on the test set. The training process employed a ten-fold group cross-validation technique, a strategy that iterates the training and evaluation process across all folds. This approach ensures that the available data is utilized comprehensively, with the model accuracy from each fold being averaged to determine the model performance. To enhance the model performance, the model hyperparameters were tuned to identify the optimal ML configuration via Bayesian optimization [64]. Bayesian optimization has been found to be more efficient than traditional random and full grid search methods as it develops a probabilistic model that maps hyperparameters to a performance metric, facilitating a strategic search compared to brute-force approaches [65–67]. In addition, the literature also highlights Bayesian optimization’s capability to manage continuous parameter spaces provided a finer search resolution, unlike grid-based approaches constrained by discrete parameters [68, 69]. This Bayesian optimization proceeded for 100 iterations to search for the optimal hyperparameters within the search space (Table S2). As shown in Figure S3, this Bayesian hyperparameter optimization improved the performance of all models investigated, but different models showed varying sensitivity to this process. For example, light gradient boosting machine (LightGBM) showed a marginal improvement in MAE (LogS) from 0.31 to 0.29, while the random forest (RF) model showed a more significant improvement in MAE (LogS) from 0.63 to 0.42. The hyperparameters identified for each model are summarized in Table S3.

**Fig. 3:**
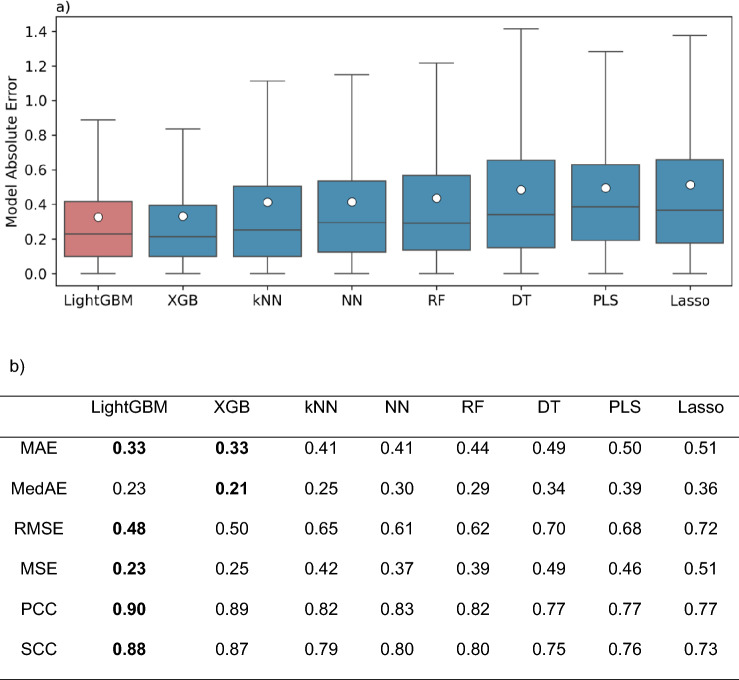
**a** Illustrates the distribution of absolute error between experimental values and predictions for eight models, based on evaluations with the test set. Each boxplot highlights the mean absolute error (MAE) and median absolute error (MedAE) using white circles and black lines, respectively. Outliers are not included for clarity, and the detailed plots including outliers are available in Figure S4. **b** Summarizes the performance of these models using six metrics: MAE, MedAE, root mean square error (RMSE), mean square error (MSE), Pearson correlation coefficient (PCC), and Spearman correlation coefficient (SCC). Within the evaluated models, the two gradient-boosted tree models, namely LightGBM and XGB, showed superior performance compared to the rest, standing out across all considered metrics

Following hyperparameter tuning, all the models configured with their optimized hyperparameters were evaluated using the test set, with their performance shown in Fig. 3. Figure 3a shows the absolute error between the model predictions and the actual targets, arranging the models in ascending order based on their MAE (LogS) from the lowest to the highest. Notably, the LightGBM and XGB models exhibited the lowest MAE, signifying superior performance relative to the other models analyzed. The performance of these models was further measured by additional model metrics as shown in Fig. 3b, where the LightGBM and XGB models consistently exceeded the performance of the other models across these metrics. This effectiveness of gradient-boosted tree models (e.g., LightGBM and XGB) aligns with literature findings on chemical tabular data within similar data regimes [23, 34, 70–72]. In addition, the accuracy associated with these models are comparable, if not better, to benchmark ML studies in the literature, where MAE values (LogS) for new solute–solvent combinations are typically between 0.4 and 0.5 [26, 27]. This performance is especially notable given that most prior studies on solubility focus on less complicated systems, such as single solvents or constant temperature. Conversely, our model was trained to predict solubility in binary solvent systems measured over a variety of temperatures.

### Prospective study

**Fig. 4:**
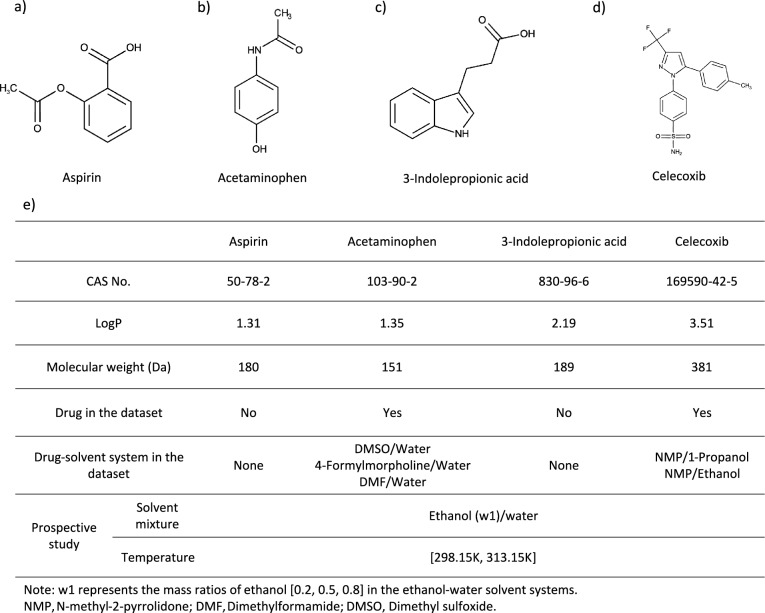
Overview of the prospective study design including four compounds: aspirin, acetaminophen, 3-indolepropionic acid, and celecoxib. **a**–**d** Show the structures of each solute. **e** Provides a summary of the compound properties, their inclusion in the dataset, and the conditions under which the solubility studies were performed

The performance of the developed LightGBM and XGB models was further assessed through an experimental prospective solubility study on four small-molecule compounds: aspirin (ASA), acetaminophen (ACM), 3-indolepropionic acid (IPA), and celecoxib (CXB). Among these compounds, ASA, ACM, and CXB are FDA-approved drugs, and IPA has been evaluated for it neuroprotective and anti-inflammatory properties for use in indications such as brain injury [73–75], stroke [76–78], and neurodegenerative diseases [79–81]. As shown in Fig. 4, these molecules were purposefully selected for their varied structural features, molecular weights, and LogP values. In addition, ACM and CXB were previously included in our dataset, while ASA and IPA represent novel solutes. The solubility measurements for these molecules were performed in ethanol/water mixtures, presenting the models with solute-binary solvent systems they had not previously encountered in the training set. To examine the effects of measurement parameters on solubility and model predictions, these studies were performed at two temperatures (298.15 and 313.15 K) and three solvent ratios (ethanol content = 0.2, 0.6, and 0.8; weight ratio). In total, 24 solubility studies were performed in triplicate as detailed in Fig. 4e.

**Fig. 5:**
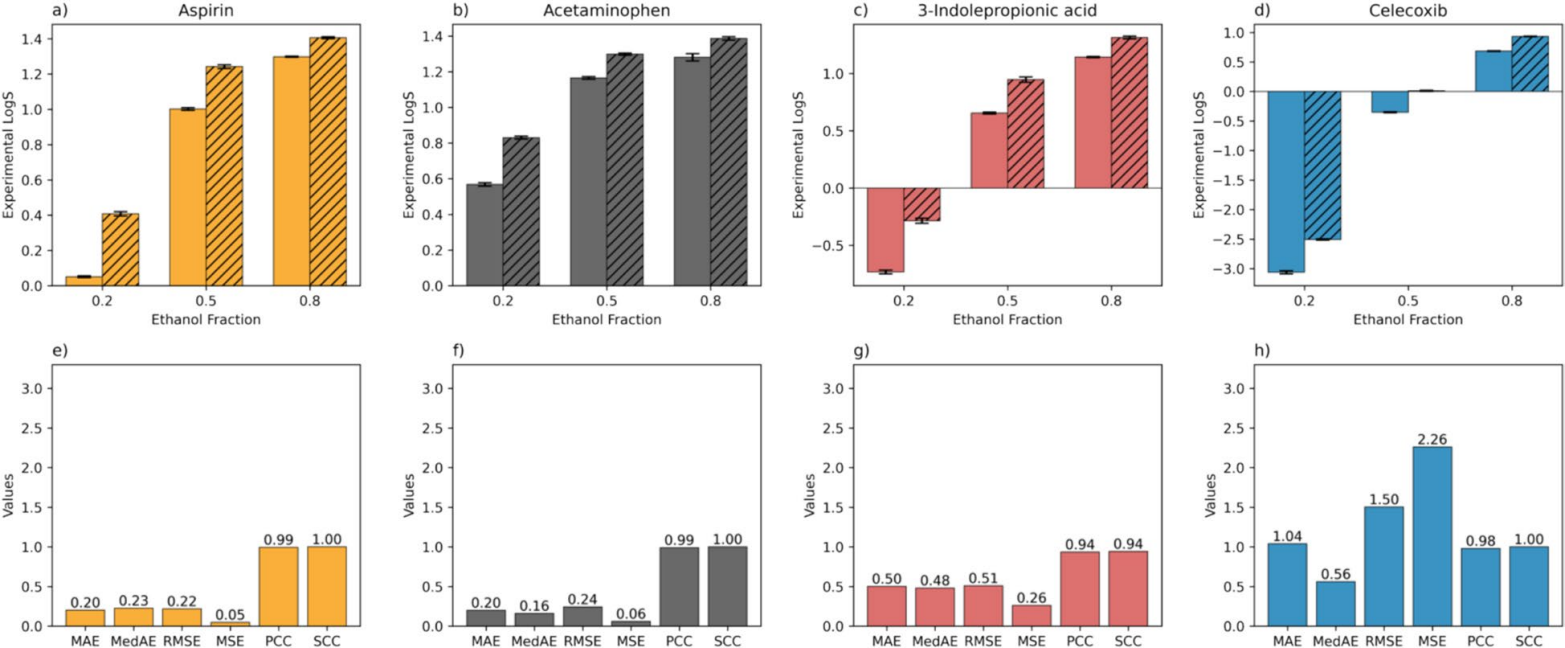
**a**–**d** The experimental solubility data for aspirin, acetaminophen, 3-indolepropinoic acid, and celecoxib in three ethanol/water mixtures (i.e., weight ratio of ethanol = 0.2, 0.5, 0.8) at temperatures of 298.15 K (non-slashed bars) and 313.15 K (slashed bars). **e**–**h** The predictive accuracy of the LightGBM model for these solubility measurements, evaluated through various metrics including mean absolute error (MAE), median absolute error (MedAE), root mean square error (RMSE), mean square error (MSE), Pearson correlation coefficient (PCC), and Spearman correlation coefficient (SCC)

The solubility measurement results are shown in Fig. 5a to d, where slashed and non-slashed bars represent results at higher (313.15 K) and lower (298.15 K) temperatures. The solubility values measured spanned from − 3 to 1.5 LogS (S in g/100 g), which is consistent with the range observed in the dataset. As expected, the results show that solubility measurement parameters significantly influence solute solubility, with an increase in solubility resulting from a rise in temperature or increase in ethanol content in the solvent mixture. Following the solubility measurements, the models’ ability to predict solubility for these solutes was evaluated, using six metrics to assess accuracy for each compound. For the LightGBM model, Fig. 5e to g indicate that the LightGBM model is effective at predicting solubility for ASA, ACM, and IPA, with MAE of 0.20, 0.20, and 0.50, respectively. The model’s ability to accurately predict the solubility of ACM may be due to its presence in the training dataset. Although ACM’s solubility in ethanol/water mixtures was not included in the dataset, the data on ACM’s solubility in other solvents likely contributed to the model’s proficiency in handling new combinations of solute and solvent. Despite ASA and IPA being new additions to the model, their predicted solubilities were notably accurate and on par with the top-performing models described in existing research, which have focused on solubility in single solvents under both constant and variable temperature conditions [26, 27]. This accuracy in prediction could stem from the fact that most of the key features for ASA and IPA fall within the scope of the dataset (to be detailed in the following paragraph). Such alignment enables the model to effectively generalize to these two new compounds, leveraging the diverse and extensive range of features present in the existing dataset which encompasses more than 100 distinct solutes.

**Fig. 6:**
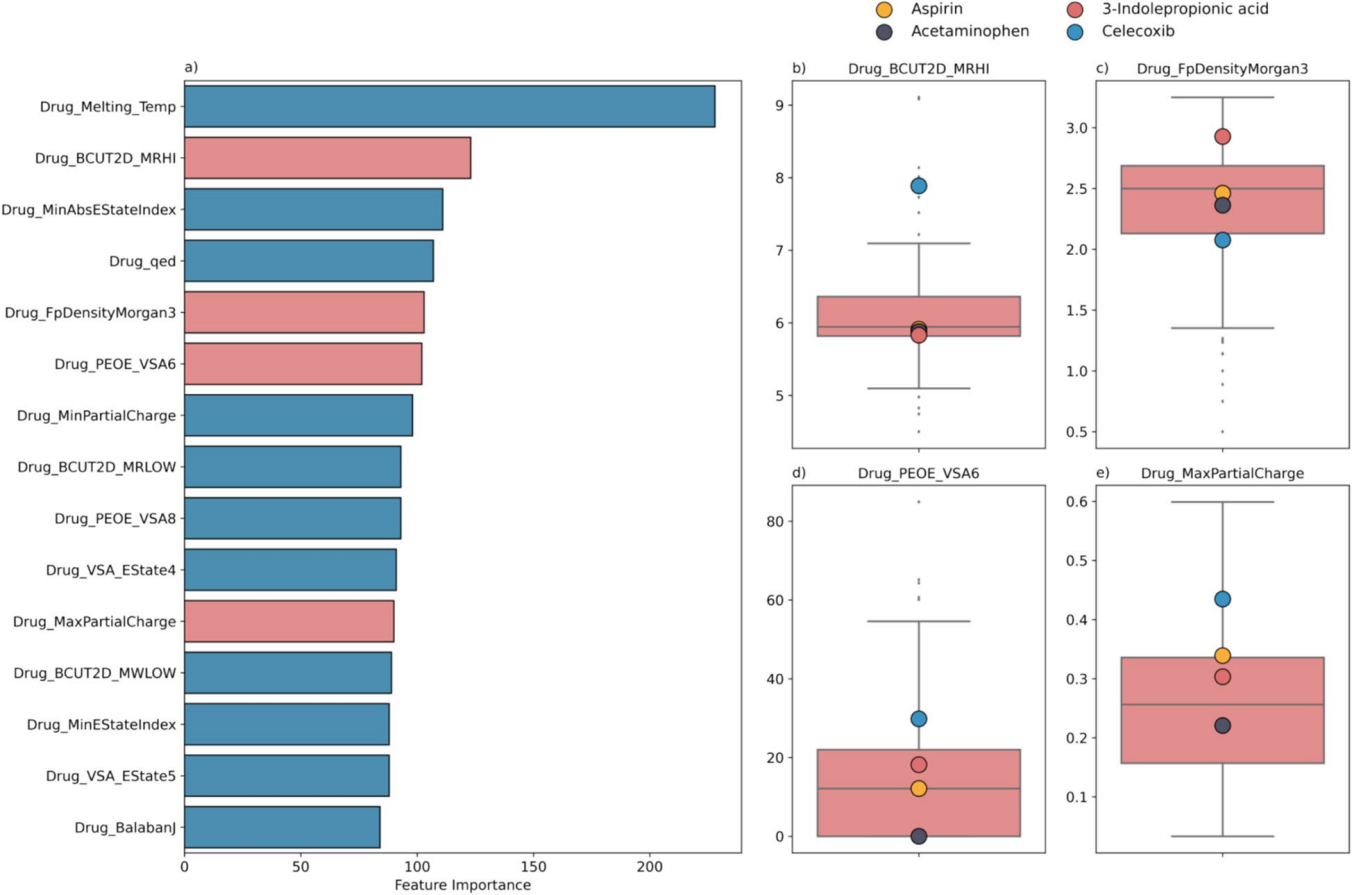
**a** The ranking of the top 15 most important solute features identified by the LightGBM model, ordered by their importance from top to bottom, with representative features for further analysis highlighted in red. **b**–**e** Comparison of the values of these features for the four compounds evaluated in the prospective study, juxtaposed against the distribution of these features across all solutes in the dataset, providing a visual depiction of their alignment or deviation from the dataset range

However, the LightGBM model’s predictions for CXB were less accurate despite CXB being present in the dataset. To explore the underlying reasons for this discrepancy, we examined the top 15 solute features deemed most influential to the LightGBM model’s decisions (Fig. 6a). The values for these features for the four compounds (ASA, ACM, IPA, and CXB) were then plotted compared with overall distribution of all the solutes within the dataset. As shown in Figure S6, it was observed that CXB deviates significantly from the dataset’s distribution in over half of (8 out of 15) of these crucial features, either falling below the first quantile, exceeding the third quantile, or being considered outliers. We attribute the worse predictive performance of the model on CXB due to higher levels of deviation from the training set in the feature space than ASA (6 of out 15), ACM (1 out of 15), and IPA (5 out of 15). The deviation of the four compounds from the dataset was further quantified by calculating their Mahalanobis distances, as discussed in Sect. “Model interpretation to understand prediction results and model limitations”.

Considering the comparable performance of the LightGBM and XGB models during the development stage, a parallel analysis was performed for the XGB model. This included both validation and interpretation with respect to critical solute attributes. During model validation, the XGB model’s performance mirrored that of the LightGBM model, demonstrating improved accuracy in predicting the solubilities of ASA, ACM, and IPA compared to CXB, with a similar accuracy level to LightGBM (difference in MAE for LogS within 0.15). Further examination of the feature distribution highlighted by XGB revealed a pattern consistent with the analysis of the LightGBM model, indicating higher deviation in CXB for most of the critical features relative to ASA, ACM, and IPA. These results are summarized in Figures S7, S8, S9, and S10.

## Discussion

### The effects of melting temperature on model performance

The melting temperature of a compound is crucial for understanding and predicting its solubility. This has been well-established in the literature with many of solubility equations [82, 83] and ML models [84, 85] developed based on the melting temperature. Similarly, in this study, melting temperature was also found to be one of the most significant features as indicated by the feature importance analysis (Fig. 6). However, the inclusion of melting temperature can also limit the application of the models as the melting temperature of a compound is not always available, which can be a common issue in fields where new compound synthesis is a primary focus.

To address this limitation, in addition to the models developed using solute melting temperatures collected from public sources, we also evaluated the performance of models developed using predicted solute melting temperatures as well as models developed omitting solute melting temperature as an input feature. Given the nature of our dataset, the predicted solute melting temperatures were generated using an open-access model [86] specifically designed for drug-like compound melting temperature prediction. These predicted melting temperatures were compared to those reported by public sources, and the chosen melting temperature model achieved a MAE of approximately 30 K, which is aligned with the accuracy of this type of model, as reported in the literature [87–90]. As shown in Table S4, models trained with both collected and predicted melting temperatures performed comparably and outperformed models that did not include solute melting temperatures. For example, the LightGBM model trained with collected and predicted melting temperatures showed an MAE for ASA LogS within 0.2, while those trained without melting points had a significantly higher MAE at 0.66 (Table S4). These results show that although melting temperature is crucial for predicting solubility accurately, using predicted melting temperatures can achieve comparable predictive accuracy. For applications where using predicted melting temperatures is necessary, it is recommended that users employ a model specifically developed for their compound type to achieve more accurate melting point predictions, and then use those predicted melting points as an input feature to generate solubility predictions using the solubility models developed in this study.

### Featurizing solutes and solvents with descriptors

Feature engineering aims to create and identify meaningful features that effectively represent the dataset, thereby improving the performance of models. This step is crucial in ML as it directly impacts the model’s ability to make accurate predictions [91, 92]. One commonly used feature engineering approach for molecular structures is to convert them into numerical features to facilitate model training [93–95]. In this study, this was done by generating RDKit descriptors and MACCS fingerprints for solutes and solvents based on their structures. These features enable the ML models to differentiate between various solutes and solvents effectively. The adoption of these computational descriptors has seen broad application in various fields, including drug discovery [96–99] and materials science [25, 100–106]. In addition to their prevailing usage, the rationale for selecting MACCS molecular fingerprints and RDKit features was also attributed to their interpretability. These descriptors offer either direct associations with specific molecular structures or yield computational molecular properties.

To evaluate whether additional features could improve model accuracy, more solute/solvent descriptors, primarily including 3D descriptors and quantum mechanics (QM)-based descriptors as listed in Table S1, were incorporated into the dataset to develop the models using the same workflow. However, as shown in Table S4, these additional features generally reduced model performance rather than improving accuracy. For example, when using the collected melting temperatures to train the models, the resulting XGB model showed a 0.2 to 0.3 increase in MAE for LogS, indicating a decrease in model performance with these additional features compared to models trained without them (Table S1). This observation aligns with existing literature, indicating that these more complex descriptors do not always enhance model performance [107–110]. For example, it was reported that 3D descriptors usually do not perform as well as 2D descriptors in quantitative structure–activity relationship (QSAR) and ML applications for molecular representations [107]. Moreover, these descriptors generally require greater computational power for feature generation and can complicate model interpretation. Consequently, these features were not further explored in this research. Nonetheless, recognizing that the impact of these features can vary depending on the problem, the methods for generating them have been included in the method section and published code to support their future application.

### Model interpretation to understand prediction results and model limitations

ML models are often seen as black boxes because the process they use to transform input data into output predictions is typically complex and not easily interpretable [111, 112]. However, interpreting ML models is important for understanding their performance and building trust in the model’s accuracy. In this study, feature importance analysis was performed as an interpretative tool to understand the deviation in the model accuracy for CXB compared to ASA, ACM, and IPA. This analysis demonstrated that the high-importance features which describe CXB deviated significantly from those of most solutes in the dataset. For instance, Fig. 6c shows that most solutes in the dataset have a BCUT2D_MRHI value ranging from 5.5 to 6.5, with ASA, ACM, and IPA fitting well within this range, whereas CXB stands out as an outlier. The BCUT2D represents a group of 2D molecular topology descriptors that have been employed in the literature as input features for solubility modeling [113–116]. Upon identifying this deviation in CXB’s featurization, where it presents as a molecule near the bounds of the high importance descriptors, we performed clustering analysis to further quantify the distribution of feature values in the dataset. All the solutes in the dataset were clustered using k-means clustering based on the solute descriptors used for model development. As shown in Table S5, eight clusters were identified, with more than half of the solutes falling into the two largest clusters. Next, the Mahalanobis distance was computed between the four tested compounds (ASA, ACM, IPA, and CXB) and all the eight clusters. The results showed that CXB significantly deviates from the two main clusters, with distances ranging from 8 to 10, almost double compared to the other three drugs, which ranged from 4 to 6. This indicates that CXB is relatively distant from the solutes currently in the dataset compared to the other three compounds, which are predicted with better accuracy. Thus, the applicability domain of the developed models corresponds to the predominant type of solutes in the dataset, which are primarily of lower molecular weight and relatively high aqueous solubility.

However, the dominance of these types of solutes also highlights one of the limitations of our dataset. Drugs that are approved and currently under development have a wide range of physico-chemical properties and thus are not fully represented by the molecules in our dataset. In response to this limitation and to further expand model applicability, we are openly sharing the dataset and source code. This is designed to foster additional research and innovation, with the hope that other research groups will build upon our dataset, thereby broadening its scope to encompass a more extensive range of drug properties. Such expansion is anticipated to refine the models’ predictive accuracy across a more diverse array of compounds. By making these resources available [117], we aim to catalyze advancements in the field, encouraging collaborative efforts to overcome existing challenges and push the boundaries of what is currently achievable in predictive modeling.

## Conclusion

In conclusion, this study reports the development, optimization, and validation of ML models for predicting the solubility of drugs in binary solvent mixtures at different temperatures. To construct this model, a comprehensive dataset containing 27,000 solubility entries was sourced from the literature. This dataset was further enriched with the melting temperatures of solutes and solvents, as well as computational descriptors. A subset of this dataset was utilized to train and optimize several ML models through Bayesian hyperparameter optimization. Among the investigated models, those demonstrating superior performance on the test set were further validated via prospective solubility studies using FDA-approved drugs and a compound that has been shown to have therapeutic potential. The validation results highlight the potential of the developed models for predicting drug solubility in given binary solvent mixtures and under varying temperature conditions. To advance research in the area and encourage enhancements to the models, the dataset, models, and source code used in this study have been made available. This initiative seeks to inspire future work aimed at expanding the dataset’s comprehensiveness and improving the model’s utility for a broader spectrum of compounds.

## Methods

### Data collection

The dataset for this study was collected via a literature review conducted using the Web of Science database and keywords “solubility” and “binary system”. Search results were limited to research articles published since 2019. Each paper was manually reviewed to ensure relevance and clarity in reporting necessary experimental parameters for solubility measurement, resulting in a total of 125 relevant papers published by different research groups. Information was extracted from these papers including the names of solutes and solvents, solvent ratios, temperatures, and solubility values. In this dataset, the two solvents in the binary mixtures were designated as “Solvent 1” and “Solvent 2”. For a specific drug-binary solvent system, “Solvent 1” refers to the solvent in a binary mixture that has a higher Pearson correlation with the solubility of the solute compared to “Solvent 2”. Solvent ratios, reported in the literature as either molar or mass ratios, were interconverted based on the reported values and the molecular weights of the solvents. Solubility values, originally reported in mole percent or weight percent, were converted to standardized LogS (S in g/100 g) for consistency with previous research. SMILES strings and melting temperatures of the solutes and solvents were obtained from the open-access databases (Chemical Abstracts Service [47], PubChem [118], Wikipedia [119], DrugBank [120], ChemSpider [121], ChemBK [122], Pesticide Properties Database [123], and ChemSrc [124]) as well as supplier websites (LKT Labs [125], Chemical Book [126], Sigma [127], Santa Cruz Biotechnology [128], Fisher Scientific [129], AK Scientific [130], Moltus Research Laboratories [131], TCI America [132], GuideChem [133], ECHEMI [134], and EBCLink [135]). To ensure accuracy of the collected melting temperature, each melting temperature was collected from three different sources, and their averages were used for modeling. In addition, for evaluating model performance when solute melting temperatures are unavailable, predicted melting temperatures were generated using the OCHEM Predictor [48] based on the models reported by Tetko et al. [86].

### Data preprocessing and splitting

The collected dataset was then preprocessed to check for duplicates, conflicting values, and ensure compound structural standardization. After examining the entire dataset with Python, around 1300 data points (less than 5% of the total dataset) were identified as duplicates; in these cases, the first data entry was retained, and subsequent duplicates were removed. These duplicates occurred when the same solubility data was reported in different tables within the same paper for comparison purposes, so no conflicting results were found. For structural standardization, tautomers and charge states were standardized using the rdMolStandardize module in RDKit. Additionally, possible diastereomers were reviewed to ensure that the collected structures accurately represented the specific diastereomers reported in the literature.

This preprocessed dataset was split into training and test sets using a group-based dataset splitting method via GroupShuffleSplit. The parameter ‘group’ was set as the solute-binary solvent systems to ensure that the same solute-binary solvent systems measured at different solvent ratios or temperatures were not repeated across the training and test sets. The training set (75%) was used for feature engineering and model development while the test set (25%) was used for model evaluation.

### Feature engineering

Solutes and solvents in the preprocessed dataset were featurized using MACCS molecular fingerprints and RDKit features for the solute and solvents, which were calculated using the RDKit package [49]. To evaluate the effects of an expanded feature set, additional features including 3D descriptors, molecular volume, dipole moment, dielectric constant, and QM-based descriptors were also generated for comparison. Specifically, molecular volume and dipole moment were computed using the RDKit package [49], solvent dielectric constant was collected from public databases [136–138], 3D descriptors were generated using rdkit.Chem.Descriptors3D module [139], and QM-based descriptors were generated using the Morfeus package [140]. A full list of these descriptors is included in Table S1. The features were then refined by removing those with no variability (variance = 0) and those that were highly correlated with others (Pearson correlation coefficient > 0.8).

### ML model training and hyperparameter optimization

A panel of eight ML models was investigated, including the light gradient boosting machine (LightGBM), extreme gradient boosting (XGB), k-nearest neighbors (kNN), neural network (NN), random forest (RF), decision tree (DT), linear regression with least absolute shrinkage and selection operator (Lasso) regularization, and partial least squares (PLS). The implementation of all models was carried out using the Scikit-learn library [141], except for LightGBM and XGB, which were developed using the LightGBM [142] and XGB [143] packages, respectively. The optimization of the models’ hyperparameters was conducted using Bayesian optimization, facilitated by the BayesSearchCV function from the Scikit-Optimize library [64]. This hyperparameter optimization process entailed a ten-fold group cross-validation strategy, executed for 100 iterations to search for the optimal set of model hyperparameters. Same as the dataset splitting method (Sect. “Data preprocessing and splitting”), the parameter ‘group’ was set as the solute-binary solvent systems. Model development was performed on a Mac mini with an 8-core Apple M1 chip and 8 GB of RAM. With this computer, the majority of models (kNN, PLS, Lasso, NN, DT) take less than one hour to complete each hyperparameter optimization, while other models (RF, LightGBM, and XGB) take three to five hours each.

### ML model evaluation

Following the optimization of hyperparameters, the models were evaluated to identify the top performers. This involved generating solubility predictions for the test set, where the predictive accuracy of each model was determined by comparing the predictions to experimental values collected from the literature. For this assessment, six ML metrics were implemented: mean absolute error (MAE), median absolute error (MedAE), root mean square error (RMSE), mean square error (MSE), Pearson correlation coefficient (PCC), and Spearman correlation coefficient (SCC). The formulas for these metrics are as follows.

1 $$\text{MAE}=\frac{{\sum }_{\text{i}=1}^{\text{N}}\left|{\text{y}}_{\text{i}}-{\widehat{y}}_{\text{i}}\right|}{\text{N}}$$

2 $$\text{MedAE}=\text{median}(\left|{\text{y}}_{\text{i}}-{\widehat{y}}_{\text{i}}\right|)$$

3 $$\text{RMSE}=\sqrt{\frac{{\sum }_{\text{i}=1}^{\text{N}}{({\text{y}}_{\text{i}}-{\widehat{y}}_{\text{i}})}^{2}}{\text{N}}}$$

4 $$\text{MSE}=\frac{{\sum }_{\text{i}=1}^{\text{N}}{({\text{y}}_{\text{i}}-{\widehat{y}}_{\text{i}})}^{2}}{\text{N}}$$

5 $$\text{PCC}=\frac{\text{cov}\left(\text{X},\text{Y}\right)}{{\upsigma }_{X}{\upsigma }_{Y}}$$

6 $$\text{SCC}=\frac{\text{cov}\left(\text{R}\left(\text{X}\right),\text{R}(\text{Y})\right)}{{\upsigma }_{\text{R}(\text{X})}{\upsigma }_{\text{R}(\text{Y})}}$$

### ML model feature importance analysis

Following the model evaluation using the test set, feature importance of the top-performing models was computed to understand the underlying factors contributing to its predictive capability. Specifically, the identified models (i.e., LightGBM and XGB) with their optimized hyperparameters were trained on the entire dataset. The importance of each feature was computed from the trained model using the built-in feature importance function in the LightGBM [144] and XGB [143] packages.

### Cluster analysis

To better understand the dataset and the model prediction results, solutes in the dataset were clustered. This process involved standardizing the solute features used for modeling with sklearn.preprocessing.StandardScaler [145], followed by clustering using sklearn.cluster.KMeans [146]. Subsequently, the Mahalanobis distance between these identified clusters and the four compounds (ASA, ACM, IPA, and CXB) in the prospective study was computed using scipy.spatial.distance.mahalanobis [147].

### Prospective study

#### Materials

Aspirin (acetylsalicylic acid, ASA, ≥ 99.0%), acetaminophen (ACM, ≥ 99.0%), 3-indolepropionic acid (IPA, 99%), acetic acid (≥ 99.0%), and trifluoroacetic acid (TFA, 99%) were purchased from Sigma Aldrich (ON, CA). Celecoxib (CXB, > 98.0%) was purchased from TCI Chemicals (Japan). Ethanol (anhydrous) was purchased from Commercial Alcohols (ON, CA). Acetonitrile (ACN, HPLC grade) was purchased from Fisher Chemical (ON, CA). Methanol (HPLC grade) was purchased from Caledon Laboratories (ON, CA). Ammonium acetate (≥ 97.0%) was purchased from BioShop (ON, CA).

#### Solubility measurement

To test the model performance prospectively, the solubility of four drug or drug-like compounds (ASA, ACM, IPA, and CXB) was measured in ethanol–water mixtures with varying solvent ratios and temperatures. Ethanol–water binary mixtures were prepared in three different ratios, with ethanol present as 20%, 50%, or 80% by weight of the mixture (measured by PB303-S balance, Mettler Toledo). For each compound, an excess of solute was added to each solvent mixture, sealed, and stored in darkness overnight. Each solubility experiment was performed in triplicates (n = 3) to ensure reproducibility and accuracy of the results. Two temperatures, 298.15 K and 313.15 K, were maintained using an incubator (INCU-Line^®^, VWR) to examine their effects on solubility. After the incubation period, the entire mixture (containing dissolved and undissolved solute) was then filtered using a syringe filter (0.22 µm pore size, polyvinylidene fluoride, Millex^®^, Sigma Aldrich) to separate the solution from the undissolved. The filtrates (i.e., saturated solution) were appropriately diluted with ethanol for high-performance liquid chromatography (HPLC) analysis to determine their concentrations.

#### HPLC analysis

The concentrations of ASA, ACM, IPA, and CXB were measured via HPLC using the Agilent Technologies 1260 Infinity II system. ACM was measured using a Restek Raptor ARC-18 column (150 mm × 4.6 mm, 2.7 µm). ASA, IPA, and CXB were measured using an Eclipse XDB-C18 column (150 mm × 4.6 mm, 5 µm). The detailed HPLC parameters are summarized in Table S6.

## Supplementary Information

Additional file 1.

## Data Availability

The dataset, codes, and models that support the findings of this study are available at the GitHub (https://github.com/Christine-Allen-Lab/Solubility_ML).
