# Supplementary material for: Towards the prediction of drug solubility in binary solvent mixtures at various temperatures using machine learning
Source: J Cheminform. 2024 Oct 28;16:117. doi: 10.1186/s13321-024-00911-3 (PMC11520512; doi:10.1186/s13321-024-00911-3)
Supplement: Supplementary file 1 — Additional file 1. [file 13321_2024_911_MOESM1_ESM.docx]

**Towards the Prediction of Drug Solubility in Binary Solvent Mixtures at Various Temperatures Using Machine Learning**

Zeqing Bao^1^, Gary Tom^234^, Austin Cheng^234^, Jeffrey Watchorn^5^, Alán Aspuru-Guzik^23456789^, Christine Allen^157^*

^1^Leslie Dan Faculty of Pharmacy, University of Toronto, Toronto, ON M5S 3M2, Canada

^2^Department of Chemistry, University of Toronto, Toronto, ON M5S 3H6, Canada

^3^Department of Computer Science, University of Toronto, Toronto, ON M5S 2E4, Canada

^4^Vector Institute for Artificial Intelligence, Toronto, ON M5S 1M1, Canada

^5^Acceleration Consortium, Toronto, ON M5S 3H6, Canada

^6^Lebovic Fellow, Canadian Institute for Advanced Research (CIFAR), Toronto, ON M5S 1M1, Canada

^7^Department of Chemical Engineering & Applied Chemistry, University of Toronto, Toronto, ON M5S 3E5, Canada

^8^Department of Materials Science & Engineering, University of Toronto, Toronto, ON M5S 3E4, Canada

^9^CIFAR Artificial Intelligence Research Chair, Vector Institute, Toronto, ON M5S 1M1, Canada

*Corresponding author: Christine Allen ([cj.allen@utoronto.ca](mailto:cj.allen@utoronto.ca))

Leslie Dan Faculty of Pharmacy, University of Toronto, Toronto, ON M5S 3M2, Canada

| **Table S1.** List of features used for dataset augmentation in this study. | | | | | | | |
| --- | --- | --- | --- | --- | --- | --- | --- |
| Features | Source | Features | Source | Features | Source | Features | Source |
| Maccs 1 to 166 | MACCS github | Chi0 | RDKit | PEOE_VSA8 | RDKit | EState_VSA4 | RDKit |
| MaxAbsEStateIndex | RDKit | Chi0n | RDKit | PEOE_VSA9 | RDKit | EState_VSA5 | RDKit |
| MaxEStateIndex | RDKit | Chi0v | RDKit | SMR_VSA1 | RDKit | EState_VSA6 | RDKit |
| MinAbsEStateIndex | RDKit | Chi1 | RDKit | SMR_VSA10 | RDKit | EState_VSA7 | RDKit |
| MinEStateIndex | RDKit | Chi1n | RDKit | SMR_VSA2 | RDKit | EState_VSA8 | RDKit |
| qed | RDKit | Chi1v | RDKit | SMR_VSA3 | RDKit | EState_VSA9 | RDKit |
| SPS | RDKit | Chi2n | RDKit | SMR_VSA4 | RDKit | VSA_EState1 | RDKit |
| MolWt | RDKit | Chi2v | RDKit | SMR_VSA5 | RDKit | VSA_EState10 | RDKit |
| HeavyAtomMolWt | RDKit | Chi3n | RDKit | SMR_VSA6 | RDKit | VSA_EState2 | RDKit |
| ExactMolWt | RDKit | Chi3v | RDKit | SMR_VSA7 | RDKit | VSA_EState3 | RDKit |
| NumValenceElectrons | RDKit | Chi4n | RDKit | SMR_VSA8 | RDKit | VSA_EState4 | RDKit |
| NumRadicalElectrons | RDKit | Chi4v | RDKit | SMR_VSA9 | RDKit | VSA_EState5 | RDKit |
| MaxPartialCharge | RDKit | HallKierAlpha | RDKit | SlogP_VSA1 | RDKit | VSA_EState6 | RDKit |
| MinPartialCharge | RDKit | Ipc | RDKit | SlogP_VSA10 | RDKit | VSA_EState7 | RDKit |
| MaxAbsPartialCharge | RDKit | Kappa1 | RDKit | SlogP_VSA11 | RDKit | VSA_EState8 | RDKit |
| MinAbsPartialCharge | RDKit | Kappa2 | RDKit | SlogP_VSA12 | RDKit | VSA_EState9 | RDKit |
| FpDensityMorgan1 | RDKit | Kappa3 | RDKit | SlogP_VSA2 | RDKit | FractionCSP3 | RDKit |
| FpDensityMorgan2 | RDKit | LabuteASA | RDKit | SlogP_VSA3 | RDKit | HeavyAtomCount | RDKit |
| FpDensityMorgan3 | RDKit | PEOE_VSA1 | RDKit | SlogP_VSA4 | RDKit | NHOHCount | RDKit |
| BCUT2D_MWHI | RDKit | PEOE_VSA10 | RDKit | SlogP_VSA5 | RDKit | NOCount | RDKit |
| BCUT2D_MWLOW | RDKit | PEOE_VSA11 | RDKit | SlogP_VSA6 | RDKit | NumAliphaticCarbocycles | RDKit |
| BCUT2D_CHGHI | RDKit | PEOE_VSA12 | RDKit | SlogP_VSA7 | RDKit | NumAliphaticHeterocycles | RDKit |
| BCUT2D_CHGLO | RDKit | PEOE_VSA13 | RDKit | SlogP_VSA8 | RDKit | NumAliphaticRings | RDKit |
| BCUT2D_LOGPHI | RDKit | PEOE_VSA14 | RDKit | SlogP_VSA9 | RDKit | NumAromaticCarbocycles | RDKit |
| BCUT2D_LOGPLOW | RDKit | PEOE_VSA2 | RDKit | TPSA | RDKit | NumAromaticHeterocycles | RDKit |
| BCUT2D_MRHI | RDKit | PEOE_VSA3 | RDKit | EState_VSA1 | RDKit | NumAromaticRings | RDKit |
| BCUT2D_MRLOW | RDKit | PEOE_VSA4 | RDKit | EState_VSA10 | RDKit | NumHAcceptors | RDKit |
| AvgIpc | RDKit | PEOE_VSA5 | RDKit | EState_VSA11 | RDKit | NumHDonors | RDKit |
| BalabanJ | RDKit | PEOE_VSA6 | RDKit | EState_VSA2 | RDKit | NumHeteroatoms | RDKit |
| BertzCT | RDKit | PEOE_VSA7 | RDKit | EState_VSA3 | RDKit | NumRotatableBonds | RDKit |

| **Table S1** (continued). | | | | | | | |
| --- | --- | --- | --- | --- | --- | --- | --- |
| Features | Source | Features | Source | Features | Source | Features | Source |
| NumSaturatedCarbocycles | RDKit | fr_alkyl_carbamate | RDKit | fr_lactone | RDKit | fr_urea | RDKit |
| NumSaturatedHeterocycles | RDKit | fr_alkyl_halide | RDKit | fr_methoxy | RDKit | xtb_ea | Morfeus |
| NumSaturatedRings | RDKit | fr_allylic_oxid | RDKit | fr_morpholine | RDKit | xtb_global_descriptor | Morfeus |
| RingCount | RDKit | fr_amide | RDKit | fr_nitrile | RDKit | xtb_homo | Morfeus |
| MolLogP | RDKit | fr_amidine | RDKit | fr_nitro | RDKit | xtb_ip | Morfeus |
| MolMR | RDKit | fr_aniline | RDKit | fr_nitro_arom | RDKit | xtb_lumo | Morfeus |
| fr_Al_COO | RDKit | fr_aryl_methyl | RDKit | fr_nitro_arom_nonortho | RDKit | Volume | RDKit |
| fr_Al_OH | RDKit | fr_azide | RDKit | fr_nitroso | RDKit | Dipole Moment | RDKit |
| fr_Al_OH_noTert | RDKit | fr_azo | RDKit | fr_oxazole | RDKit | Dielectric constant | Public database |
| fr_ArN | RDKit | fr_barbitur | RDKit | fr_oxime | RDKit | PMI1 | RdKit_3D |
| fr_Ar_COO | RDKit | fr_benzene | RDKit | fr_para_hydroxylation | RDKit | PMI2 | RdKit_3D |
| fr_Ar_N | RDKit | fr_benzodiazepine | RDKit | fr_phenol | RDKit | PMI3 | RdKit_3D |
| fr_Ar_NH | RDKit | fr_bicyclic | RDKit | fr_phenol_noOrthoHbond | RDKit | NPR1 | RdKit_3D |
| fr_Ar_OH | RDKit | fr_diazo | RDKit | fr_phos_acid | RDKit | NPR2 | RdKit_3D |
| fr_COO | RDKit | fr_dihydropyridine | RDKit | fr_phos_ester | RDKit | RadiusOfGyration | RdKit_3D |
| fr_COO2 | RDKit | fr_epoxide | RDKit | fr_piperdine | RDKit | InertialShapeFactor | RdKit_3D |
| fr_C_O | RDKit | fr_ester | RDKit | fr_piperzine | RDKit | Eccentricity | RdKit_3D |
| fr_C_O_noCOO | RDKit | fr_ether | RDKit | fr_priamide | RDKit | Asphericity | RdKit_3D |
| fr_C_S | RDKit | fr_furan | RDKit | fr_prisulfonamd | RDKit | SpherocityIndex | RdKit_3D |
| fr_HOCCN | RDKit | fr_guanido | RDKit | fr_pyridine | RDKit | PBF | RdKit_3D |
| fr_Imine | RDKit | fr_halogen | RDKit | fr_quatN | RDKit |  |  |
| fr_NH0 | RDKit | fr_hdrzine | RDKit | fr_sulfide | RDKit |  |  |
| fr_NH1 | RDKit | fr_hdrzone | RDKit | fr_sulfonamd | RDKit |  |  |
| fr_NH2 | RDKit | fr_imidazole | RDKit | fr_sulfone | RDKit |  |  |
| fr_N_O | RDKit | fr_imide | RDKit | fr_term_acetylene | RDKit |  |  |
| fr_Ndealkylation1 | RDKit | fr_isocyan | RDKit | fr_tetrazole | RDKit |  |  |
| fr_Ndealkylation2 | RDKit | fr_isothiocyan | RDKit | fr_thiazole | RDKit |  |  |
| fr_Nhpyrrole | RDKit | fr_ketone | RDKit | fr_thiocyan | RDKit |  |  |
| fr_SH | RDKit | fr_ketone_Topliss | RDKit | fr_thiophene | RDKit |  |  |
| fr_aldehyde | RDKit | fr_lactam | RDKit | fr_unbrch_alkane | RDKit |  |  |


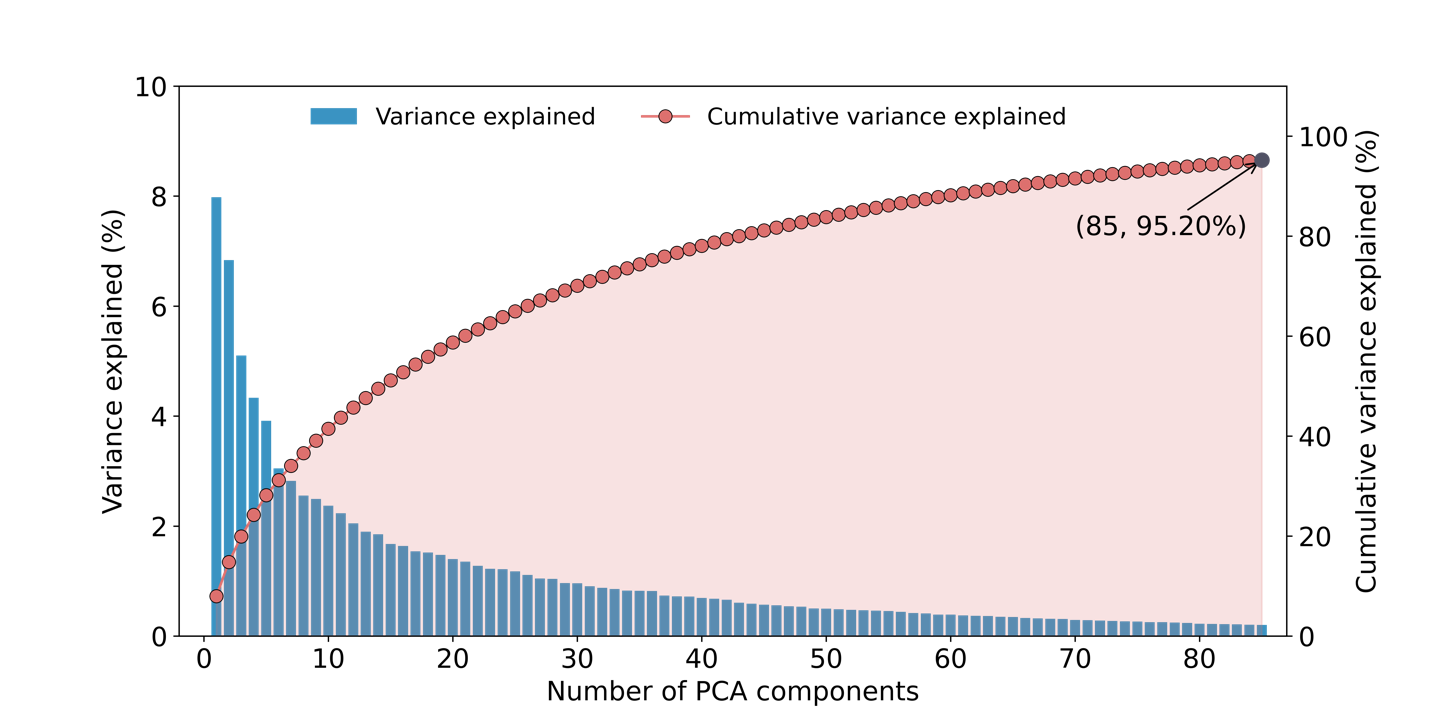


**Figure S1.** The graph illustrates the variance explained by principal components in a principal component analysis (PCA). Each bar represents the percentage of total variance in the dataset accounted for by an individual principal component, ordered by their significance. The cumulative percentage of variance explained is shown by the line graph with connected circles. The graph highlights that 85 principal components together explain a significant portion of the information in the dataset (>95%).


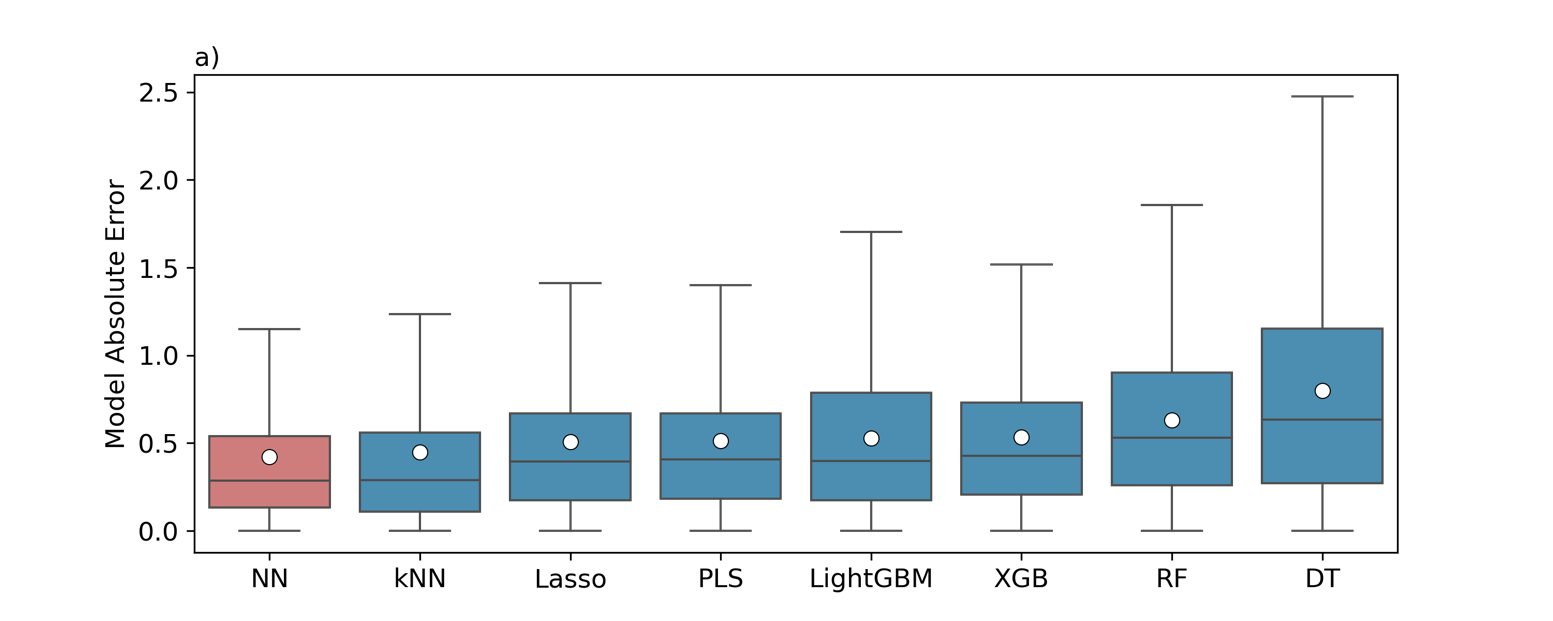


| b) |  |  |  |  |  |  |  |  |
| --- | --- | --- | --- | --- | --- | --- | --- | --- |
|  | NN | kNN | Lasso | PLS | LightGBM | XGB | RF | DT |
| MAE | 0.413 | 0.426 | 0.541 | 0.512 | 0.509 | 0.538 | 0.618 | 0.753 |
| MedAE | 0.298 | 0.245 | 0.413 | 0.401 | 0.404 | 0.419 | 0.523 | 0.606 |
| RMSE | 0.591 | 0.672 | 0.734 | 0.668 | 0.661 | 0.709 | 0.773 | 0.974 |
| MSE | 0.349 | 0.451 | 0.539 | 0.446 | 0.437 | 0.503 | 0.598 | 0.948 |
| PCC | 0.83 | 0.785 | 0.718 | 0.77 | 0.78 | 0.733 | 0.764 | 0.471 |
| SCC | 0.825 | 0.752 | 0.717 | 0.74 | 0.742 | 0.698 | 0.72 | 0.442 |

**Figure S2.** A panel of ML models were trained using the dataset refined based on principal component analysis (85 components). (a) Illustrates the distribution of absolute errors between experimental values and predictions for eight models, based on evaluations with the test subset. Each boxplot highlights the mean absolute error (MAE) and median absolute error (MedAE) using white circles and black lines, respectively. (b) Summarizes the performance of these models using six metrics: MAE, MedAE, root mean square error (RMSE), mean square error (MSE), Pearson correlation coefficient (PCC), and Spearman correlation coefficient (SCC).

| **Table S2.** A summary of the model hyperparameter search space. | |
| --- | --- |
| **Model** | **Hyperparameters** |
| DT | {'max_depth': Integer(low=3, high=20, prior='uniform', transform='identity'), 'splitter': Categorical(categories=('best', 'random'), prior=None), 'min_samples_split': Real(low=0.01, high=0.1, prior='uniform', transform='identity'), 'min_samples_leaf': Integer(low=1, high=20, prior='uniform', transform='identity'), 'max_features': Categorical(categories=('auto', 'sqrt', 'log2'), prior=None)} |
| RF | {'n_estimators': Integer(low=10, high=400, prior='uniform', transform='identity'), 'max_depth': Integer(low=3, high=20, prior='uniform', transform='identity'), 'min_samples_split': Real(low=0.01, high=0.1, prior='uniform', transform='identity'), 'min_samples_leaf': Integer(low=1, high=20, prior='uniform', transform='identity'), 'max_features': Categorical(categories=('auto', 'sqrt', 'log2'), prior=None), 'bootstrap': Categorical(categories=(True, False), prior=None)} |
| XGB | {'n_estimators': Integer(low=10, high=400, prior='uniform', transform='identity'), 'learning_rate': Real(low=0.01, high=0.3, prior='log-uniform', transform='identity'), 'max_depth': Integer(low=3, high=20, prior='uniform', transform='identity'), 'subsample': Real(low=0.5, high=1.0, prior='uniform', transform='identity'), 'colsample_bytree': Real(low=0.5, high=1.0, prior='uniform', transform='identity'), 'gamma': Real(low=0, high=5, prior='uniform', transform='identity')} |
| NN | {'hidden_layer_sizes': Integer(low=2, high=64, prior='uniform', transform='identity'), 'alpha': Real(low=0.0001, high=0.1, prior='log-uniform', transform='identity'), 'learning_rate_init': Real(low=0.001, high=0.1, prior='log-uniform', transform='identity'), 'activation': Categorical(categories=('relu', 'tanh', 'logistic'), prior=None)} |
| LightGBM | {'num_leaves': Integer(low=10, high=400, prior='uniform', transform='identity'), 'max_depth': Integer(low=3, high=20, prior='uniform', transform='identity'), 'learning_rate': Real(low=0.01, high=0.3, prior='log-uniform', transform='identity'), 'n_estimators': Integer(low=100, high=1000, prior='uniform', transform='identity'), 'bagging_fraction': Real(low=0.5, high=1, prior='uniform', transform='identity'), 'feature_fraction': Real(low=0.5, high=1, prior='uniform', transform='identity'), 'min_child_samples': Integer(low=5, high=100, prior='uniform', transform='identity')} |
| Lasso | {'alpha': Real(low=0.0001, high=1, prior='log-uniform', transform='identity'), 'selection': Categorical(categories=('cyclic', 'random'), prior=None)} |
| kNN | {'n_neighbors': Integer(low=2, high=50, prior='uniform', transform='identity'), 'weights': Categorical(categories=('uniform', 'distance'), prior=None), 'algorithm': Categorical(categories=('auto', 'ball_tree', 'kd_tree', 'brute'), prior=None), 'leaf_size': Integer(low=10, high=100, prior='uniform', transform='identity'), 'p': Integer(low=1, high=2, prior='uniform', transform='identity')} |
| PLS | {'n_components': Integer(low=2, high=6, prior='uniform', transform='identity'), 'max_iter': Integer(low=250, high=1000, prior='uniform', transform='identity')} |


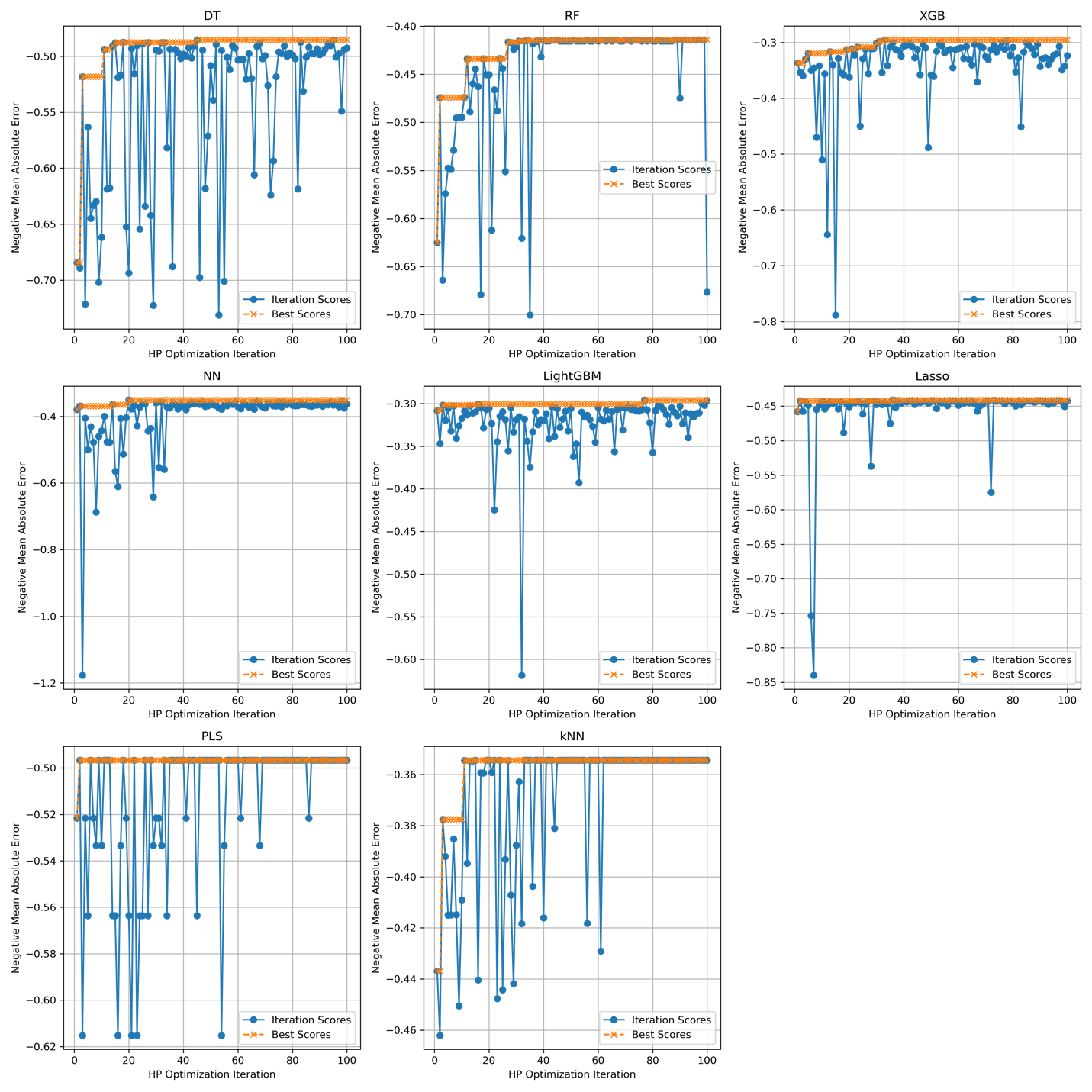


**Figure S3.** Summary of model performance over 100 iterations of Bayesian hyperparameter (HP) optimization. Each blue scatter point represents the model performance (i.e., negative mean absolute error) for an iteration, while the orange crosses indicate the best scores achieved up to that iteration.

| **Table S3.** A summary of the optimized model hyperparameters. | |
| --- | --- |
| **Model** | **Best Hyperparameter** |
| DT | {'best_estimator': DecisionTreeRegressor(max_depth=12, max_features='auto', min_samples_split=0.01,  random_state=0), 'best_params': OrderedDict([('max_depth', 12), ('max_features', 'auto'), ('min_samples_leaf', 1), ('min_samples_split', 0.01), ('splitter', 'best')]), 'best_score': -0.48415265264463914} |
| RF | {'best_estimator': RandomForestRegressor(max_depth=20, max_features='auto', min_samples_split=0.01,  n_estimators=242, n_jobs=6, random_state=0), 'best_params': OrderedDict([('bootstrap', True), ('max_depth', 20), ('max_features', 'auto'), ('min_samples_leaf', 1), ('min_samples_split', 0.01), ('n_estimators', 242)]), 'best_score': -0.4141210340471326} |
| XGB | {'best_estimator': XGBRegressor(base_score=None, booster=None, callbacks=None,  colsample_bylevel=None, colsample_bynode=None,  colsample_bytree=0.5, early_stopping_rounds=None,  enable_categorical=False, eval_metric=None, feature_types=None,  gamma=0.0, gpu_id=None, grow_policy=None, importance_type=None,  interaction_constraints=None, learning_rate=0.06469343991269094,  max_bin=None, max_cat_threshold=None, max_cat_to_onehot=None,  max_delta_step=None, max_depth=6, max_leaves=None,  min_child_weight=None, missing=nan, monotone_constraints=None,  n_estimators=400, n_jobs=6, num_parallel_tree=None, predictor=None,  random_state=0, ...), 'best_params': OrderedDict([('colsample_bytree', 0.5), ('gamma', 0.0), ('learning_rate', 0.06469343991269094), ('max_depth', 6), ('n_estimators', 400), ('subsample', 1.0)]), 'best_score': -0.2952340304563773} |
| NN | {'best_estimator': MLPRegressor(alpha=0.03216551794885616, hidden_layer_sizes=64,  learning_rate_init=0.020941350061487837, random_state=0), 'best_params': OrderedDict([('activation', 'relu'), ('alpha', 0.03216551794885616), ('hidden_layer_sizes', 64), ('learning_rate_init', 0.020941350061487837)]), 'best_score': -0.35069351619633904} |
| LightGBM | {'best_estimator': LGBMRegressor(bagging_fraction=0.5, feature_fraction=0.5,  learning_rate=0.12274969844791342, max_depth=7,  min_child_samples=100, n_estimators=1000, n_jobs=6, num_leaves=10,  random_state=0), 'best_params': OrderedDict([('bagging_fraction', 0.5), ('feature_fraction', 0.5), ('learning_rate', 0.12274969844791342), ('max_depth', 7), ('min_child_samples', 100), ('n_estimators', 1000), ('num_leaves', 10)]), 'best_score': -0.2957353050539909} |
| Lasso | {'best_estimator': Lasso(alpha=0.0010998680471010754, selection='random'), 'best_params': OrderedDict([('alpha', 0.0010998680471010754), ('selection', 'random')]), 'best_score': -0.4410236260050085} |
| kNN | {'best_estimator': KNeighborsRegressor(algorithm='kd_tree', leaf_size=100, n_jobs=6, n_neighbors=2,  p=1, weights='distance'), 'best_params': OrderedDict([('algorithm', 'kd_tree'), ('leaf_size', 100), ('n_neighbors', 2), ('p', 1), ('weights', 'distance')]), 'best_score': -0.35436202765769476} |
| PLS | {'best_estimator': PLSRegression(max_iter=444, n_components=6), 'best_params': OrderedDict([('max_iter', 444), ('n_components', 6)]), 'best_score': -0.49659831072676336} |


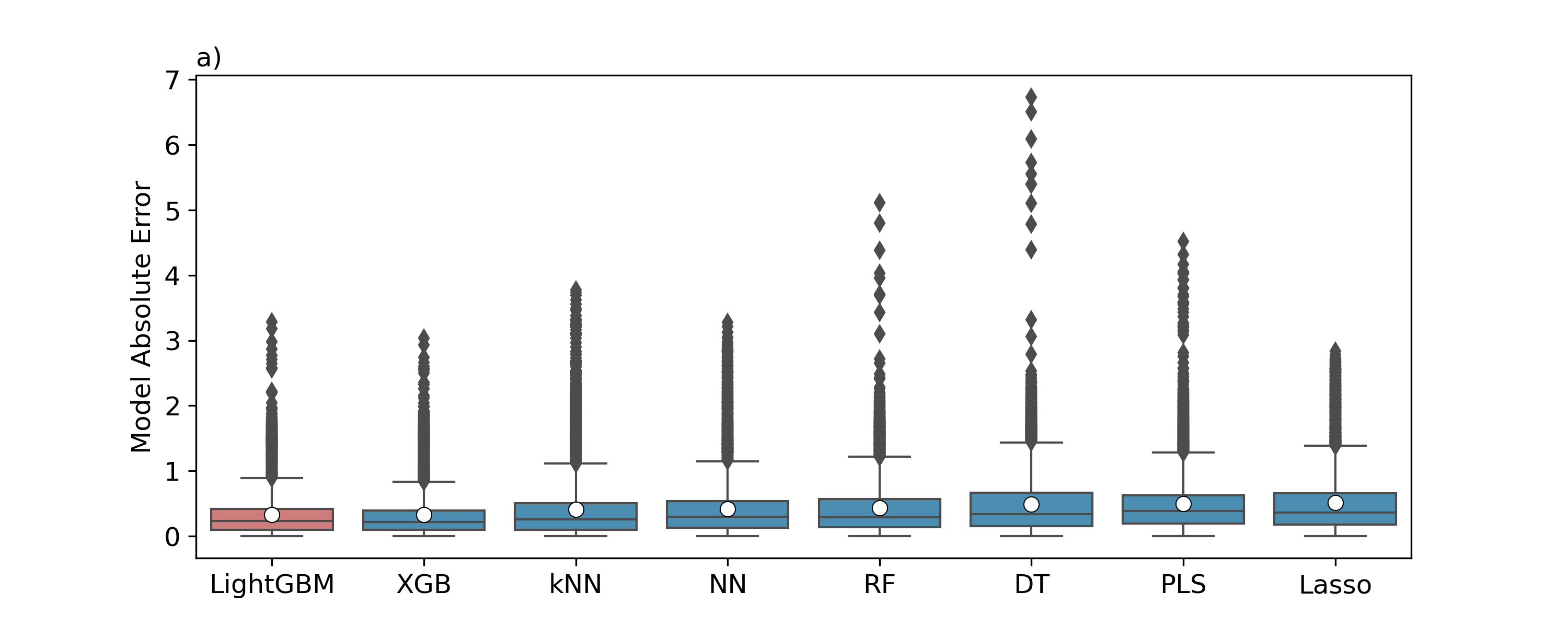


**Figure S4.** (a) Illustrates the distribution of absolute error between experimental values and predictions for eight models, based on evaluations with the test subset. Each boxplot highlights the mean absolute error (MAE), median absolute error (MedAE), and outliers using white circles, black lines, and black dots, respectively.


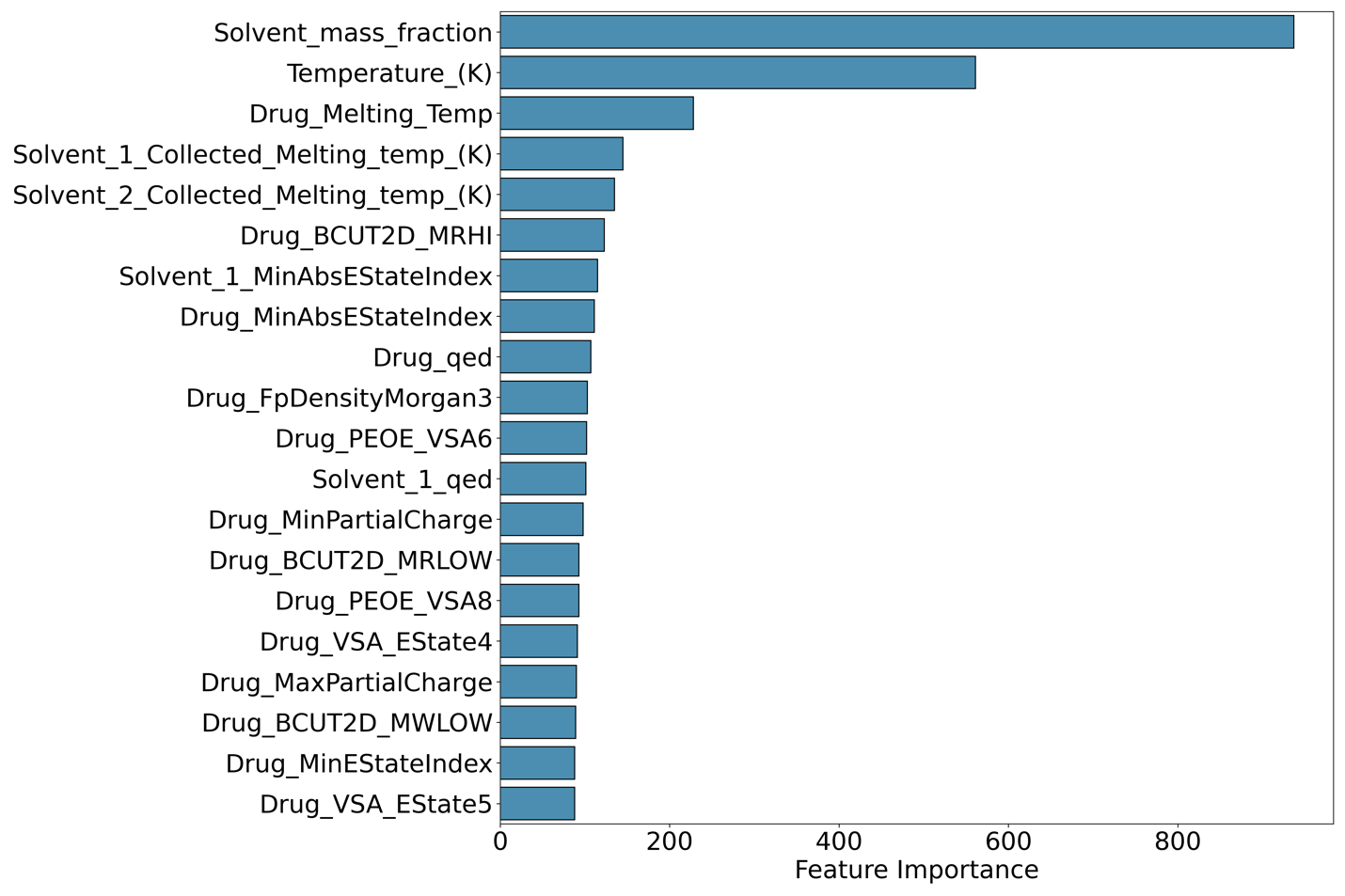


**Figure S5.** A summary of the ranking of the top 20 most important features identified by the LightGBM model, ordered by their importance from top to bottom.


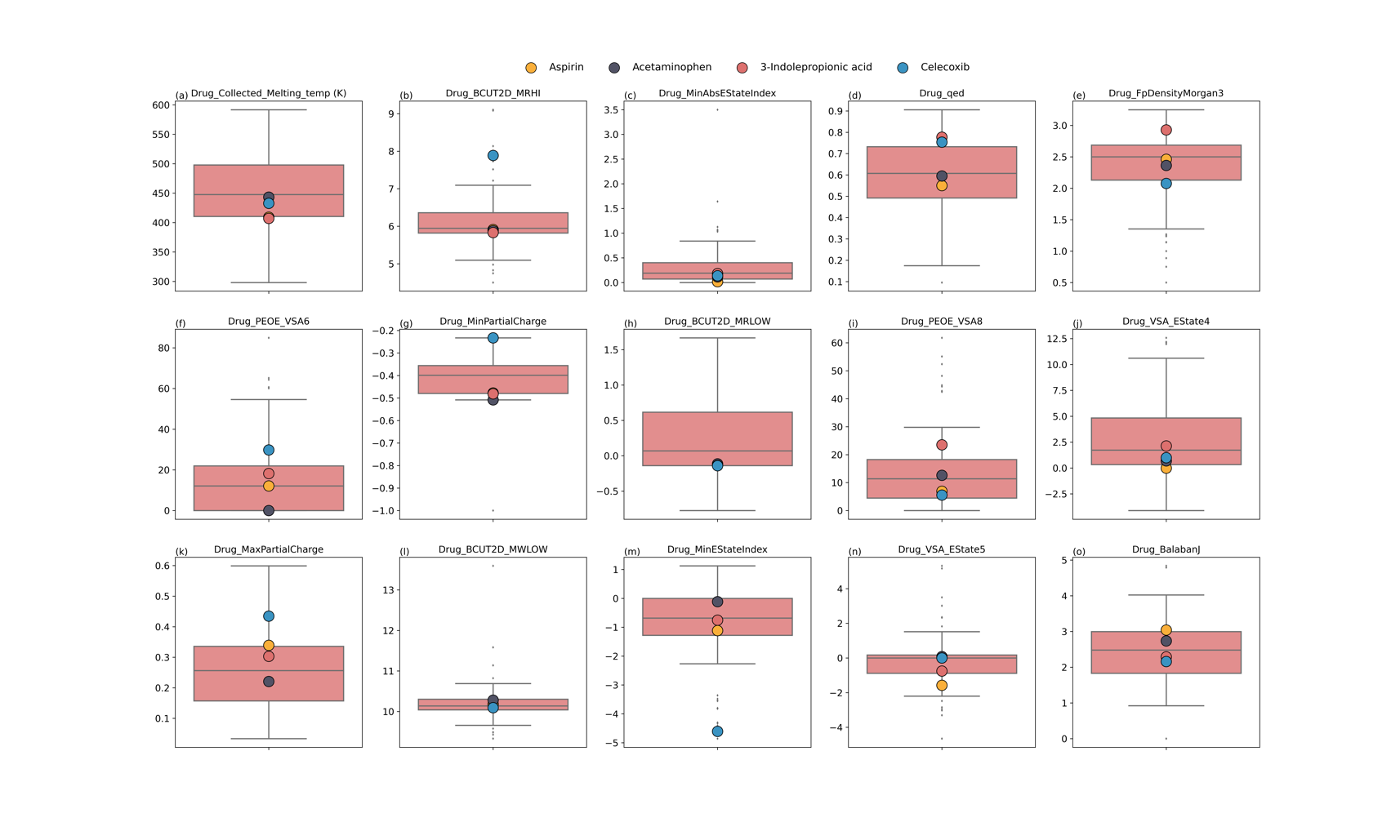


**Figure S6.** (a-o) Comparison of the top 15 solute features identified by the LightGBM model for the four compounds evaluated in the prospective study, juxtaposed against the distribution of these features across all solutes within the dataset. This includes a graphical representation to illustrate how these compounds align with or diverge from the dataset range.


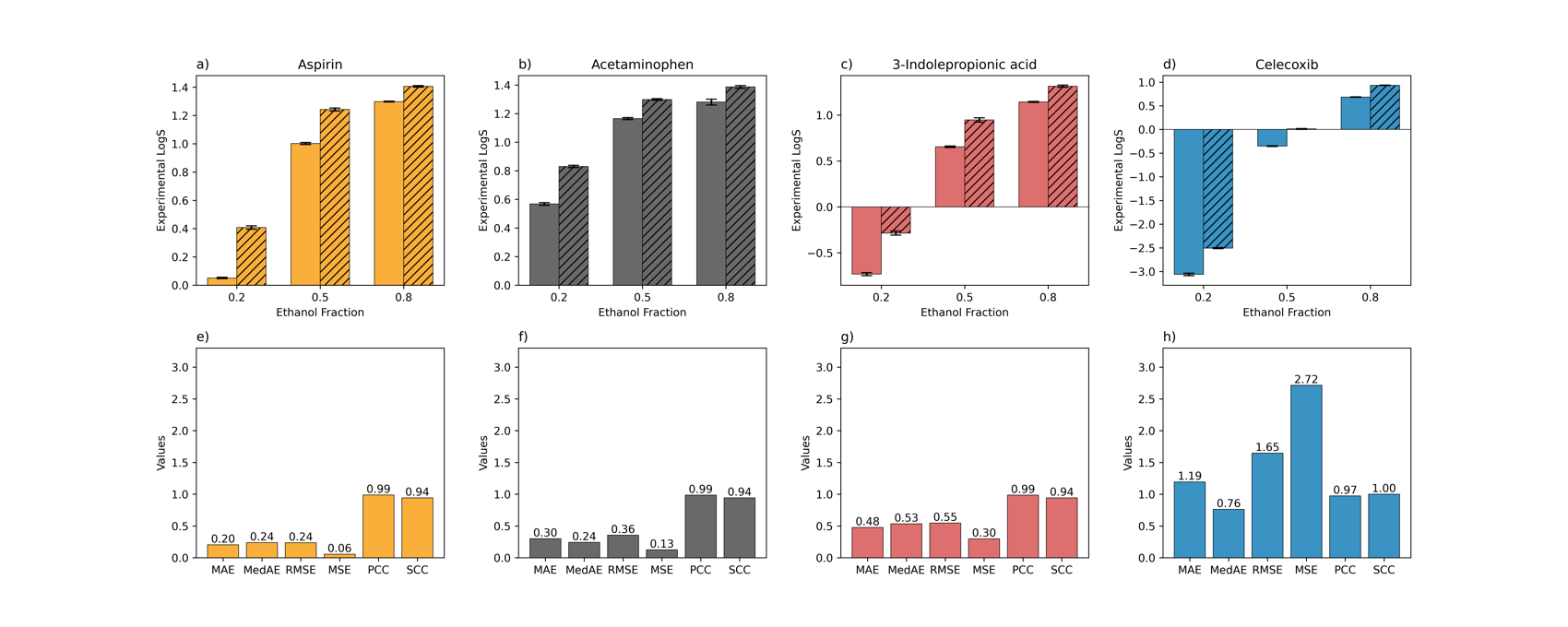


**Figure S7.** (a-d) The experimental solubility data for aspirin, acetaminophen, 3-indolepropinoic acid, and celecoxib across three ethanol/water mixture ratios at temperatures of 298.15 K (non-slashed bars) and 313.15 K (slashed bars). (e-h) The predictive accuracy of the XGB model for these solubility measurements, evaluated through various metrics including mean absolute error (MAE), median absolute error (MedAE), root mean square error (RMSE), mean square error (MSE), Pearson correlation coefficient (PCC), and Spearman correlation coefficient (SCC).


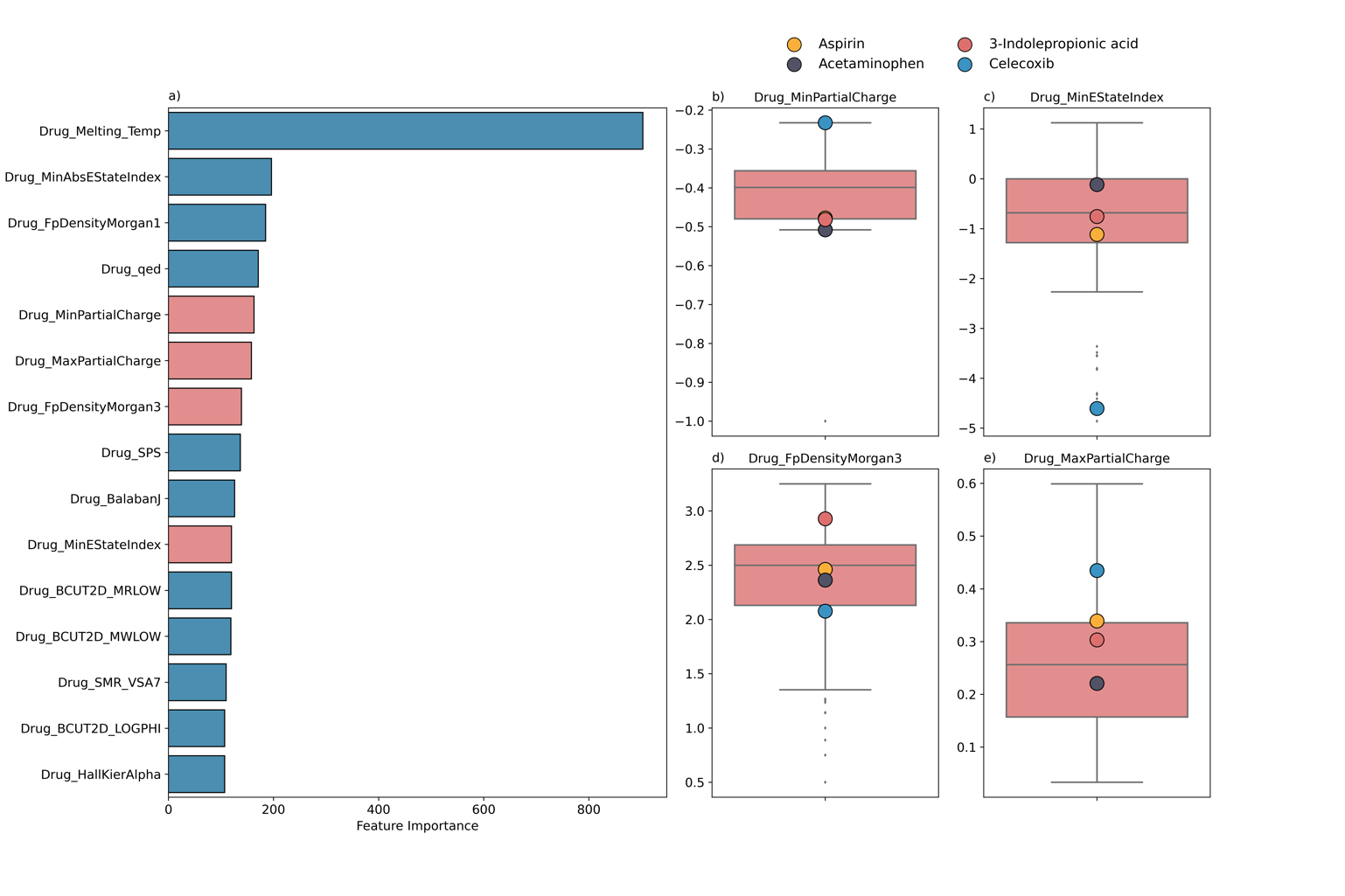


**Figure S8.** (a) The ranking of the top 15 most important solute features identified by the XGB model, ordered by their importance from top to bottom, with representative features for further analysis highlighted in red. (b-e) Comparison of the values of these features for the four compounds evaluated in the prospective study, juxtaposed against the distribution of these features across all solutes in the dataset, providing a visual depiction of their alignment or deviation from the dataset range.

**
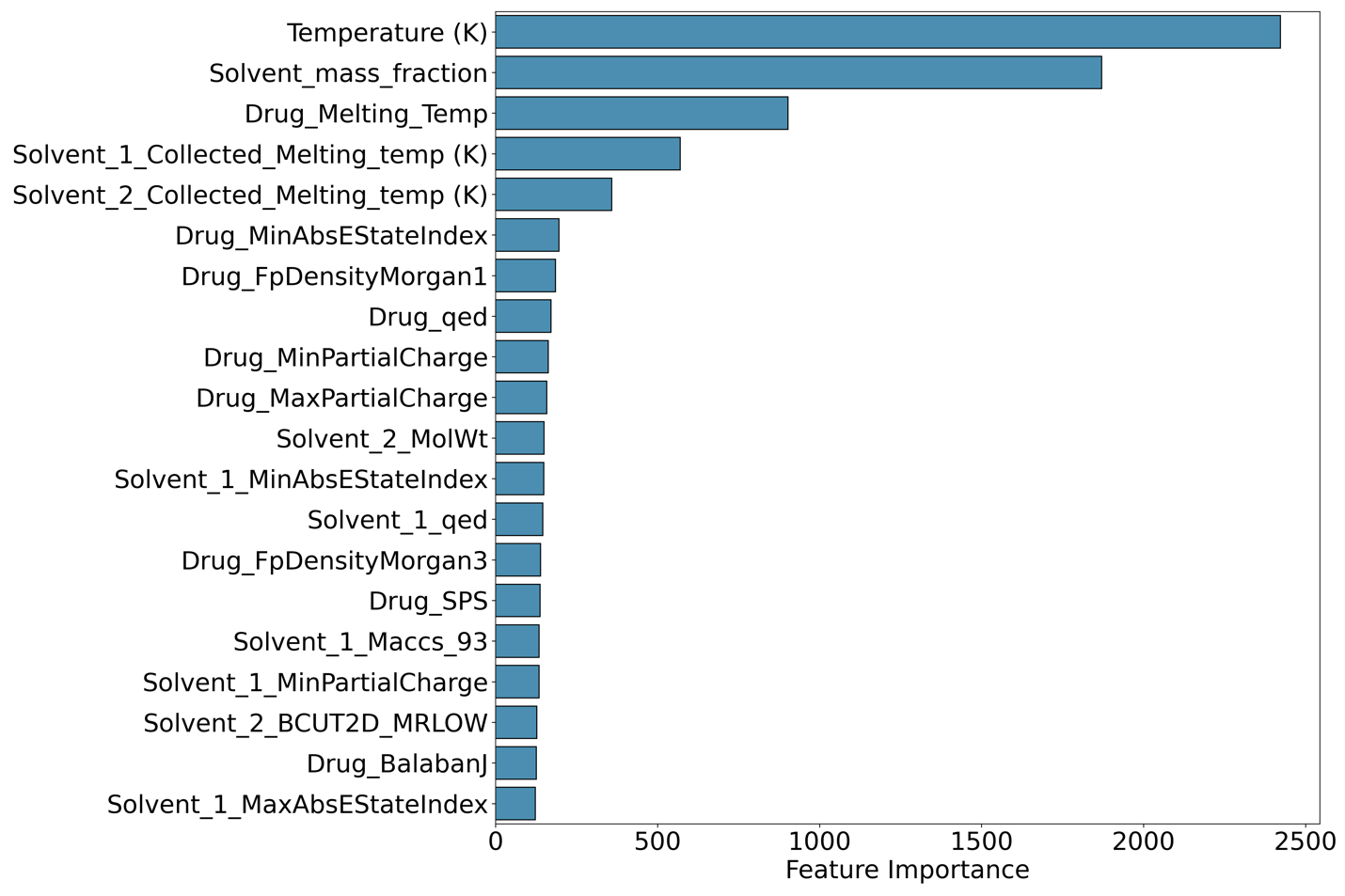
**

**Figure S9.** A summary of the ranking of the top 20 most important features identified by the XGB model, ordered by their importance from top to bottom.

**
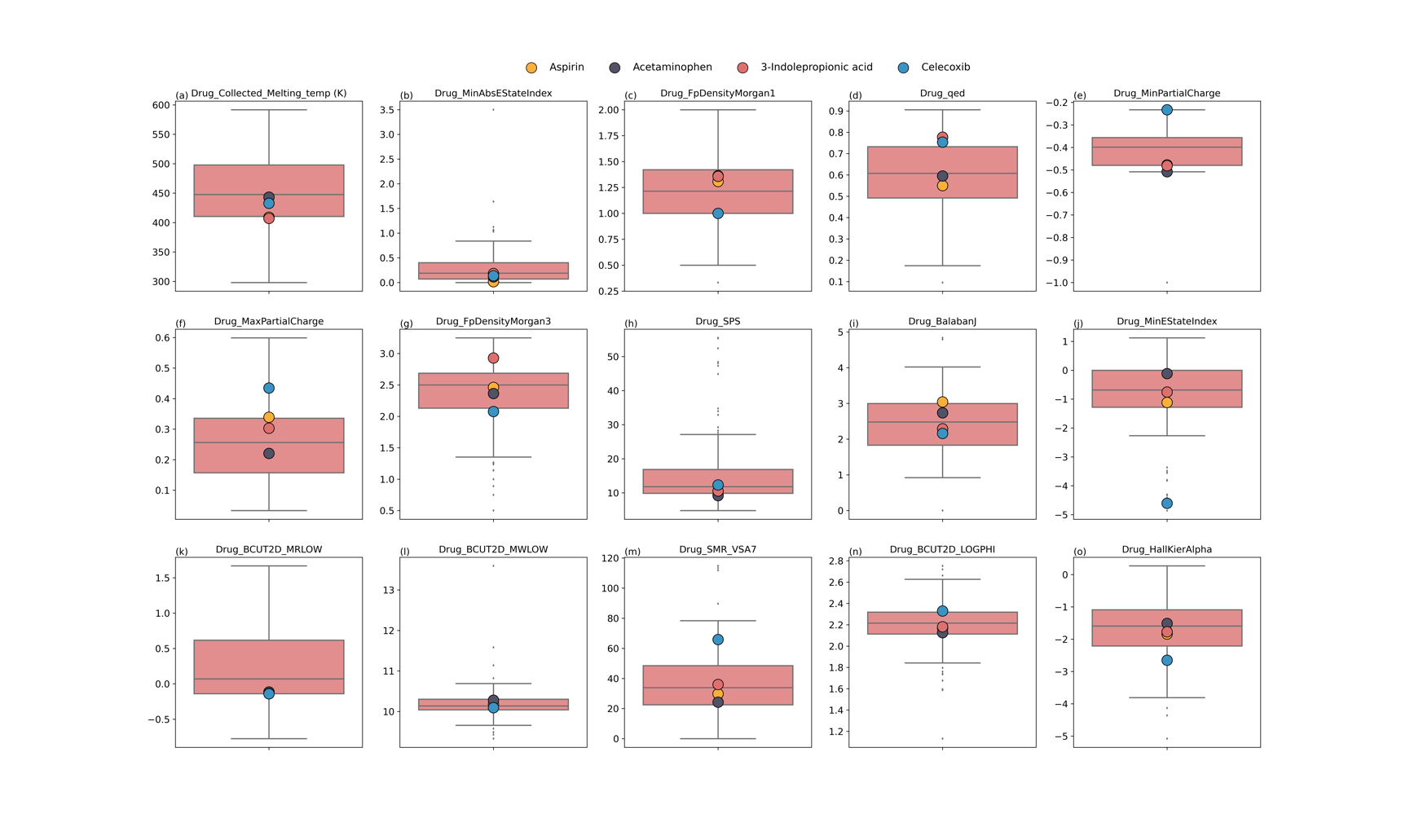
**

**Figure S10.** (a-o) Comparison of the top 15 solute features identified by the XGB model for the four compounds evaluated in the prospective study, juxtaposed against the distribution of these features across all solutes within the dataset. This includes a graphical representation to illustrate how these compounds align with or diverge from the dataset range.

| **Table S4.** Performance of LightGBM and XGB models in predicting the solubility of aspirin (ASA), acetaminophen (ACM), 3-indolepropionic acid (IPA), and celecoxib (CXB). The models are developed using six datasets, which include original or expanded features, and incorporate collected, predicted, or no solute melting temperatures. The original features include solubility measurement parameters, RDKit descriptors, and MACCS molecular fingerprints. The expanded dataset builds on the original features by including additional solute/solvent descriptors, primarily their 3D and quantum mechanics-based descriptors. A full list of these descriptors is shown in Table S1. | | | | | | | |
| --- | --- | --- | --- | --- | --- | --- | --- |
|  | | Original features | | | Expanded features | | |
| Solute melting temperature | | Collected | Predicted | Not included | Collected | Predicted | Not included |
| LightGBM (MAE, LogS) | ASA | 0.20 | 0.16 | 0.66 | 0.19 | 0.15 | 0.37 |
|  | ACM | 0.20 | 0.22 | 0.31 | 0.34 | 0.70 | 0.53 |
|  | IPA | 0.50 | 0.47 | 0.72 | 0.52 | 0.42 | 0.64 |
|  | CXB | 1.04 | 1.00 | 1.18 | 1.19 | 1.37 | 1.29 |
| XGB  (MAE, LogS) | ASA | 0.20 | 0.36 | 0.39 | 0.51 | 0.31 | 0.46 |
|  | ACM | 0.30 | 0.32 | 0.21 | 0.57 | 0.45 | 0.48 |
|  | IPA | 0.48 | 0.46 | 0.96 | 0.58 | 0.63 | 0.66 |
|  | CXB | 1.19 | 1.06 | 1.28 | 1.28 | 1.14 | 1.29 |

| **Table S5.** A summary of the Mahalanobis distances between aspirin (ASA), acetaminophen (ACM), 3-indolepropionic acid (IPA), celecoxib (CXB) and the dataset solute clusters. | | | | | |
| --- | --- | --- | --- | --- | --- |
| Cluster index | Cluster Size | Mahalanobis distance | | | |
|  |  | CXB | ACM | IPA | ASA |
| 0 | 17 | 3.05 | 3.17 | 3.21 | 2.32 |
| 1 | 34 | 8.75 | 5.42 | 5.79 | 4.57 |
| 2 | 41 | 10.53 | 6.25 | 6.24 | 5.88 |
| 3 | 6 | 1.05 | 2.02 | 1.77 | 1.79 |
| 4 | 12 | 1.11 | 1.94 | 2.75 | 2.26 |
| 5 | 6 | 4.33 | 5.53 | 5.36 | 6.04 |
| 6 | 1 | N/A | N/A | N/A | N/A |
| 7 | 6 | 2.04 | 1.65 | 4.59 | 2.75 |

| **Table S6.** HPLC parameters, including mobile phases, column temperatures, flowrates, and detection wavelengths, for the quantification of aspirin (ASA), acetaminophen (ACM), 3-indolepropionic acid (IPA), and celecoxib (CXB). | | | | |
| --- | --- | --- | --- | --- |
| Compound | ASA | ACM | IPA | CXB |
| Mobile phase A | Water  (0.1% TFA) | Water  (50 mM ammonium acetate, pH=4) | Water  (0.1% TFA) | Water |
| Mobile phase B | ACN  (0.1% TFA) | ACN | ACN  (0.1% TFA) | MeOH |
| Mobile phase ratio  (A/B, v/v) | 75/25 | 0 min: 90/10 4 min: 50/50  4.1 min: 90/10  6 min: 90/10  10 min: 90/10 | 60/40 | 20/80 |
| Column temperature  (°C) | 30 | Room temperature | 35 | Room temperature |
| Flowrate  (mL/min) | 1 | 1 | 1 | 1 |
| Detection wavelength  (nm) | 238 | 250 | 282 | 251 |
| *The salicylic acid (resulting from ASA hydrolysis) was quantified simultaneously with ASA to account for ASA hydrolysis. | | | | |
